# Structure, substrate specificity, and catalytic mechanism of human D-2-HGDH and insights into pathogenicity of disease-associated mutations

**DOI:** 10.1038/s41421-020-00227-0

**Published:** 2021-01-12

**Authors:** Jun Yang, Hanwen Zhu, Tianlong Zhang, Jianping Ding

**Affiliations:** 1 State Key Laboratory of Molecular Biology, Shanghai Institute of Biochemistry and Cell Biology, Center for Excellence in Molecular Cell Science, University of Chinese Academy of Sciences, Chinese Academy of Sciences, 320 Yue-Yang Road, Shanghai, 200031 China; 2 School of Life Science, Hangzhou Institute for Advanced Study, University of Chinese Academy of Sciences, 1 Xiangshan Road, Hangzhou, Zhejiang 310024 China

**Keywords:** Molecular biology, X-ray crystallography

## Abstract

D-2-hydroxyglutarate dehydrogenase (D-2-HGDH) catalyzes the oxidation of D-2-hydroxyglutarate (D-2-HG) into 2-oxoglutarate, and genetic D-2-HGDH deficiency leads to abnormal accumulation of D-2-HG which causes type I D-2-hydroxyglutaric aciduria and is associated with diffuse large B-cell lymphoma. This work reports the crystal structures of human D-2-HGDH in apo form and in complexes with D-2-HG, D-malate, D-lactate, L-2-HG, and 2-oxoglutarate, respectively. D-2-HGDH comprises a FAD-binding domain, a substrate-binding domain, and a small C-terminal domain. The active site is located at the interface of the FAD-binding domain and the substrate-binding domain. The functional roles of the key residues involved in the substrate binding and catalytic reaction and the mutations identified in D-2-HGDH-deficient diseases are analyzed by biochemical studies. The structural and biochemical data together reveal the molecular mechanism of the substrate specificity and catalytic reaction of D-2-HGDH and provide insights into the pathogenicity of the disease-associated mutations.

## Introduction

2-Hydroxyglutarate (2-HG) is a low-abundance metabolite that exists in normal cells of many organisms but has unknown physiological function(s) for a long time^1–5^. It has two enantiomers owing to the chiral C2 atom, namely D-2-HG and L-2-HG. Only very recently have researchers begun to uncover how 2-HG is produced and consumed in cells. A number of enzymes were demonstrated to produce 2-HG as side reactions with low catalytic efficiency^6,7^. In humans, 3-phosphoglycerate dehydrogenase can convert 2-oxyglutaric acid (2-OG or α-KG) to D-2-HG^8^. Mitochondrial hydroxyacid-oxoacid transhydrogenase catalyzes the oxidation of 4-hydroxybutyrate to succinate semialdehyde coupled with the reduction of 2-OG to D-2-HG^9,10^. Mutations of cytosolic isocitrate dehydrogenase IDH1 and mitochondrial IDH2 confer a neomorphic activity to convert 2-OG to D-2-HG^11–13^. On the other hand, both mitochondrial and cytoplasmic malate dehydrogenases and lactate dehydrogenases can catalyze the reduction of 2-OG to L-2-HG^14–16^. Regardless of their promiscuous metabolic origins, D-2-HG and L-2-HG can be converted back to 2-OG by two mitochondrial enzymes in humans, namely D-2-HG and L-2-HG dehydrogenases (D-2-HGDH and L-2-HGDH)^2,17–19^.

Although its physiological function(s) remain elusive, 2-HG has attracted great attention as abnormal accumulation of 2-HG in humans is associated with pathogeneses of a rare neurometabolic disorder called 2-hydroxyglutaric aciduria (2-HGA)^6^ and multiple types of cancers^7,11–13,20,21^. 2-HGA is a fatal autosomal recessive disease in infancy or early childhood, and is diagnosed by increased 2-HG levels in body fluid, blood, and urine. Based on the symptoms and causes, 2-HGA can be classified into several different types. D-2-HGA commonly exhibits a broad clinical neonatal symptoms including developmental delay, epilepsy, hypotonia, cardiomyopathy, and dysmorphic features, which can be further divided into two types: type I is caused by loss-of-function D-2-HGDH mutants, and type II is caused by gain-of-function IDH1 and IDH2 mutants, both of which lead to D-2-HG accumulation^13,22–24^. L-2-HGA is associated with progressive ataxia, psychomotor retardation, leukodystrophy, and brain tumors, and is caused by a deficient L-2-HGDH, which leads to L-2-HG accumulation^18,25–27^. A mixed type of 2-HGA called D,L-2-HGA is found in neonates with encephalopathy coupled with the accumulation of both D-2-HG and L-2-HG, which is caused by mutations in mitochondrial citrate carrier SLC25A1^28,29^. In addition, missense heterozygous mutations have been identified in D-2-HGDH from a cohort of diffuse large B-cell lymphoma (DLBCL) patients, which also lead to D-2-HG accumulation^30^. Mechanistically, both 2-HG enantiomers have been proposed to act as competitive inhibitors of 2-OG and thus interfere with the functions of many 2-OG-dependent enzymes, including the Jmjc-domain containing family of histone demethylases and the TET family of DNA dioxygenases. Hence, abnormal 2-HG accumulation has been implicated in malignant progression of many cancers and is emerged as an important biomarker in the diagnosis of gliomas^11–13,15^.

Human D-2-HGDH belongs to the 2-hydroxy acid dehydrogenase subfamily of the vanillyl alcohol oxidase (VAO) and *para*-cresol methylhydroxylase (PCMH) flavoprotein family, all members of which consist of a conserved FAD-binding domain and a variable substrate-binding domain^17,31^. Besides D-2-HGDH, the 2-hydroxy acid dehydrogenase subfamily also includes D-lactate dehydrogenases (D-LDHs) and glycolate oxidoreductase GlcD, which all catalyze the oxidation of 2-hydroxyl of the substrate to carbonyl using FAD as cofactor^32–34^. So far, only the structure of *E. coli* D-LDH in apo form was reported^35^. Human D-2-HGDH consists of 521 residues, which shares low sequence similarity with other members of the VAO/PCMH family. Previous biochemical studies showed that D-2-HGDH has broad substrate specificity, exhibiting high activity towards D-2-HG and substantial activity towards D-malate (D-MAL) and D-lactate (D-LAC) but no activity towards L-2-HG^17^. In addition, the enzymatic activity of D-2-HGDH can be stimulated by Zn^2+^, Co^2+^, and Mn^2+^ with the highest activity detected in the presence of Zn^2+^, suggesting that D-2-HGDH is a Zn^2+^-dependent dehydrogenase^17^. However, the structure and the molecular basis for substrate specificity and the catalytic reaction of D-2-HGDH are unknown. The functional roles of D-2-HGDH mutations in the pathogeneses of D-2-HGDH-deficient diseases remain elusive.

In this work, we report the crystal structures of human D-2-HGDH in apo form and in complexes with D-2-HG, D-MAL, D-LAC, L-2-HG, and 2-OG, respectively. Based on the structural, mutagenesis, and biochemical analyses, we identified the key residues involved in the recognition and binding of the substrate and the catalytic reaction, and uncovered the functional roles of the mutations identified in D-2-HGDH-deficient diseases. The structural and biochemical data together reveal the molecular mechanism of the substrate specificity and catalytic reaction of D-2-HGDH and provide insights into the pathogenicity of disease-associated mutations.

## Results

### Structure of human D-2-HGDH

Human D-2-HGDH was expressed in *E. coli* and purified using affinity chromatography and gel filtration chromatography (Supplementary Fig. S1). Crystallization of D-2-HGDH in the absence of any ligands yielded crystals of D-2-HGDH in apo form. Crystals of D-2-HGDH in complexes with D-2-HG, D-MAL, D-LAC, L-2-HG, and 2-OG (Supplementary Fig. S2) were obtained by soaking the apo form crystals in drops supplemented with the ligand and ZnCl_2_. Structure of the apo D-2-HGDH was solved at 2.20 Å resolution, and structures of D-2-HGDH in complexes with D-2-HG, D-MAL, D-LAC, L-2-HG, and 2-OG were solved at 2.65 Å, 2.80 Å, 2.62 Å, 3.00 Å, and 2.80 Å resolution, respectively (Table 1). The asymmetric unit contains two D-2-HGDH molecules with essentially identical conformation (Supplementary Fig. S3). In all the structures, there is a FAD non-covalently bound to the active site (Supplementary Fig. S4a), which is apparently co-purified with the enzyme, indicating that D-2-HGDH binds FAD tightly. In the apo D-2-HGDH structure, there is no metal ion or ligand bound at the active site (Supplementary Fig. S4b). In the structures of D-2-HGDH in complexes with D-2-HG, D-MAL, and D-LAC, there is unambiguous electron density for a metal ion and a D-2-HG (or D-MAL or D-LAC) at the active site (Supplementary Fig. S4c–e). In the structures of D-2-HGDH in complexes with L-2-HG and 2-OG, there is also clearly defined electron density for a metal ion and the lactate moiety of L-2-HG or pyruvate moiety of 2-OG; however, the acetate moiety of both L-2-HG and 2-OG is disordered (Supplementary Fig. S4f, g). The bound metal ion in the ligand-bound D-2-HGDH complexes is interpreted as Zn^2+^ based on the following reasons: (1) The crystals of the complexes were obtained by soaking the crystals of the apo D-2-HGDH in the crystallization drops containing ZnCl_2_; (2) The Zn^2+^ rather than other divalent metal ions is refined with reasonable B factor compared with the protein (Table 1); (3) The bound Zn^2+^ has clearly defined electron density in the simulated annealing composite omit maps at high contour level (3σ) (Supplementary Fig. S5); (4) The bond distances of Zn^2+^ with its coordinating residues, the FAD or substrate are all in proper range (Supplementary Fig. S6) and in consistent with the reported values in the literature^36^.

**Table 1:** Summary of diffraction data and structure refinement statistics.

	D-2-HGDH FAD	D-2-HGDH FAD + Zn+D-2-HG	D-2-HGDH FAD + Zn+D-MAL	D-2-HGDH FAD + Zn+D-LAC	D-2-HGDH FAD + Zn+2-OG	D-2-HGDH FAD + Zn+L-2-HG
PDB code	6LPN	6LPP	6LPQ	6LPT	6LPX	6LPU
Diffraction data						
Wavelength (Å)	0.9789	0.9789	0.9788	0.9788	0.9788	0.9792
Space group	*P*2_1_	*P*2_1_	*P*2_1_	*P*2_1_	*P*2_1_	*P*2_1_
Cell parameters						
* a*, *b*, *c* (Å)	72.3, 94.9, 72.7	72.3, 94.6, 73.4	72.6, 94.6, 73.7	72.3, 94.4, 73.6	72.2, 94.9, 73.2	71.7, 94.5, 72.8
* α*, *β*,*γ* (°)	90, 113.3, 90	90, 111.9, 90	90, 111.6, 90	90, 111.8, 90	90, 112.3, 90	90, 112.2, 90
Resolution (Å)	50.0–2.20 (2.28–2.20)^a^	50.0–2.65 (2.74–2.65)	50.0–2.80 (2.90–2.80)	50.0–2.62 (2.71–2.62)	50.0–2.80 (2.90–2.80)	50.0–3.00 (3.11–3.00)
Observed reflections	299,485	90,457	132,903	188,447	150,017	91,529
Unique reflections (I/σ(I) > 0)	45,047	26,662	21,823	27,511	22,571	19,104
Average redundancy	6.6 (5.6)	3.4 (3.2)	6.1 (5.1)	6.8 (7.0)	6.6 (6.0)	4.8 (4.4)
Average I/σ(I)	10.0 (2.0)	8.1 (1.8)	3.4 (1.0)	8.6 (2.0)	5.6 (1.3)	5.1 (1.1)
Completeness (%)	99.7 (99.9)	99.0 (98.1)	95.7 (81.8)	99.5 (99.9)	99.4 (99.9)	99.5 (97.8)
*R*_merge_ (%)^b^	26.9 (89.7)	10.4 (37.5)	29.7 (80.1)	15.4 (65.6)	28.4 (109.4)	22.0 (74.2)
CC_1/2_ (%)	93.3 (60.9)	98.4 (85.2)	93.3 (61.7)	99.0 (84.1)	96.3 (65.8)	95.0 (65.1)
Refinement and structure model						
No. of reflections (*Fo* > 0σ(*Fo*))	45,004	26,610	21,657	27,431	22,492	19,015
Working set	42,648	25,343	20,578	26,134	21,368	18,073
Test set	2356	1267	1079	1297	1124	942
*R*-factor/free *R*-factor (%)^c^	16.7/21.7	20.1/24.5	21.5/25.5	19.3/23.3	19.6/23.7	22.4/25.5
No. of atoms	7639	7159	7153	7353	7249	7155
Protein	7022	6886	7027	7027	7027	7027
Ligand	106	168	124	118	126	126
Ion	–	2	2	2	2	2
Water	511	103	–	206	94	–
Wilson B-factor (Å^2^)	26.9	33.3	34.9	32.8	36.9	41.6
Average B-factor (Å^2^)	31.3	34.6	32.5	34.3	36.9	41.6
Protein	30.9	34.7	32.6	34.5	37.0	41.7
Ligand	24.4	32.2	27.6	27.4	32.3	36.5
Ion	–	28.4	27.7	25.9	29.0	37.6
Water	38.4	28.9	–	32.1	32.0	–
RMS deviations						
Bond lengths (Å)	0.006	0.003	0.003	0.003	0.004	0.004
Bond angles (^o^)	1.06	0.810	0.844	0.876	1.055	0.986
Ramachandran plot (%)						
Favored	98.0	98.0	98.0	98.0	98.0	98.0
Allowed	2.0	2.0	2.0	2.0	2.0	2.0
Outliers	0.0	0.0	0.0	0.0	0.0	0.0

^a^Numbers in parentheses represent the highest resolution shell.

^b^*R*_merge_ = ∑_*hkl*_∑_*i* _| *I*_*i*_(*hkl*)−<*I*(*hkl*)>|/∑_*hkl*_∑_*i*_*I*_*i*_(*hkl*).

^c^*R*-factor = ∑_*hkl*_| |*F*_*o*_|−|*F*_*c*_| |/∑_*hkl* _|*F*_o_|.

It is worthy to note that D-2-HGDH did not exhibit enzymatic activity towards D-2-HG in the crystals of D-2-HGDH in complex with Zn^2+^ and D-2-HG. There are two main reasons to explain why D-2-HG is not turned over in the crystals. (1) The crystals of D-2-HGDH in complex with D-2-HG were obtained by soaking the crystals of the apo D-2-HGDH in crystallization drops supplemented with D-2-HG and ZnCl_2_ at 16 °C followed by flash-freezing and stored in liquid nitrogen (–196 °C). As the enzymatic reaction is usually temperature-sensitive, the catalytic reaction of D-2-HGDH towards D-2-HG would be very slow at 16 °C during crystal soaking or even slower at –196 °C during freezing and storage in liquid nitrogen. (2) In our enzymatic activity assay, artificial electron acceptors DCIP and PMS were employed to oxidize the reduced FAD into the oxidized FAD which is essential for the catalytic reaction. In the crystals, it would be dioxygen (O_2_) responsible for oxidation of the reduced FAD into the oxidized FAD. Previously, it was shown that the rate of electron transferring from the reduced FAD in D-2-HGDH to DCIP and PMS was 5-fold higher than that to O_2_, indicating that D-2-HGDH has a higher activity using DCIP and PMS as electron acceptors than O_2_^17^. The same reasons could also explain why D-MAL and D-LAC are not turned over in the crystals.

**Fig. 1:**
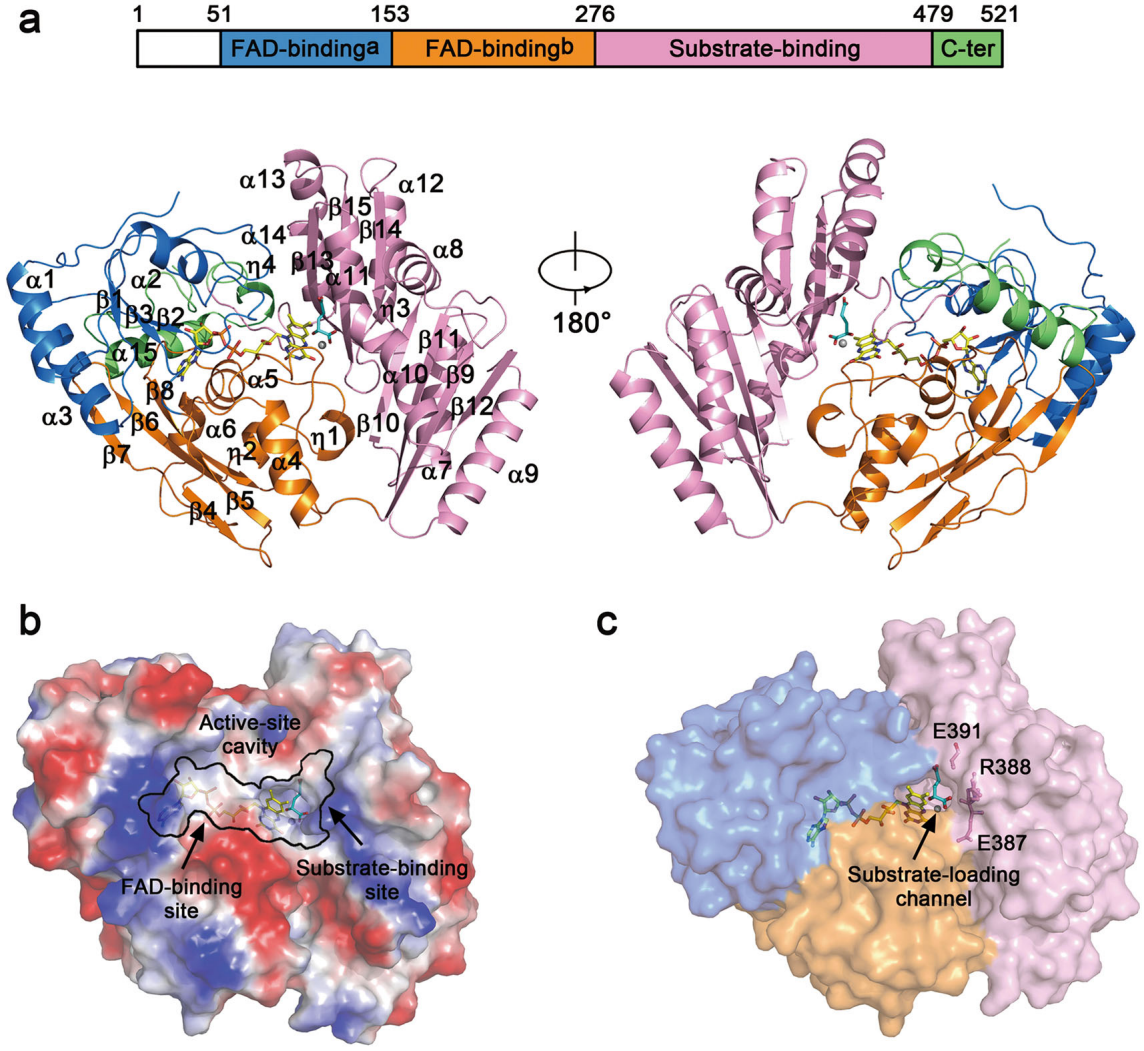
Structure of human D-2-HGDH. **a** Overall structure of D-2-HGDH in complex with FAD and D-2-HG in ribbon diagram. The color-coding scheme of the (sub)domains is shown above. The secondary structure elements are labeled. The Zn^2+^ at the active site is shown with a gray sphere, and the FAD and D-2-HG are shown with ball-and-stick models and colored in yellow and cyan, respectively. **b** Electrostatic potential surface of D-2-HGDH showing the location of the active site consisting of the FAD-binding site and the substrate-binding site. The surface charge distribution is displayed as blue for positive, red for negative, and white for neutral. **c** Molecular surface of D-2-HGDH showing the location of the substrate-loading channel linking the active site to the outside.

Like other members of the VAO/PCMH flavoprotein family^33,35^, D-2-HGDH is comprised of two major domains named the FAD-binding domain and the substrate-binding domain supplemented with a small C-terminal domain (Fig. 1a and Supplementary Fig. S7). The FAD-binding domain adopts a classical PCMH-type fold consisting of two subdomains: subdomain a (residues 51–153) composes of a three-stranded parallel β-sheet (β1–β3) surrounded by three α-helices (α1–α3), and subdomain b (residues 154–276) composes of a five-stranded antiparallel β-sheet (β4–β8) surrounded by three α-helices (α4–α6). The substrate-binding domain (residues 277–479) composes of a seven-stranded antiparallel β-sheet (β9–β15) flanked by five α-helices (α7, α9, α12, α13, and α14) on one side and three α-helices (α8, α10, and α11) on the other. The small C-terminal domain (residues 480–521) consists of an α-helix (α15) and a 3_10_ helix (η4), which packs along the FAD-binding domain to shield the FAD-binding site from the solvent.

The active site resides at the interface of the FAD-binding domain and the substrate-binding domain, and consists of the FAD-binding site and the substrate-binding site (Fig. 1a, b). It is buried about 8 Å below the protein surface and is linked to the solvent via a channel which may act as the trafficking route for the substrate into and the product out of the active site and thus is designated as “the substrate-loading channel” (Fig. 1b, c). The channel is hydrophilic in nature and consists of several charged residues, including Glu387, Arg388, and Glu391, which could interact favorably with and thus facilitate the passing of the charged substrate and product. Previously, Gygli *et al*. identified four ligand migration paths in VAO, namely the cap path, the FAD path, the subunit interface path, and the co-ligands dioxygen and hydrogen peroxide path using Monte Carlo based simulations^37^. The subunit interface was suggested to be the most likely diffusion path for the substrate^37^. Structural comparison of D-2-HGDH with VAO shows that the proposed substrate-loading channel in D-2-HGDH is structurally related to the co-ligands path in VAO (Supplementary Fig. S8).

### FAD binding

**Fig. 2:**
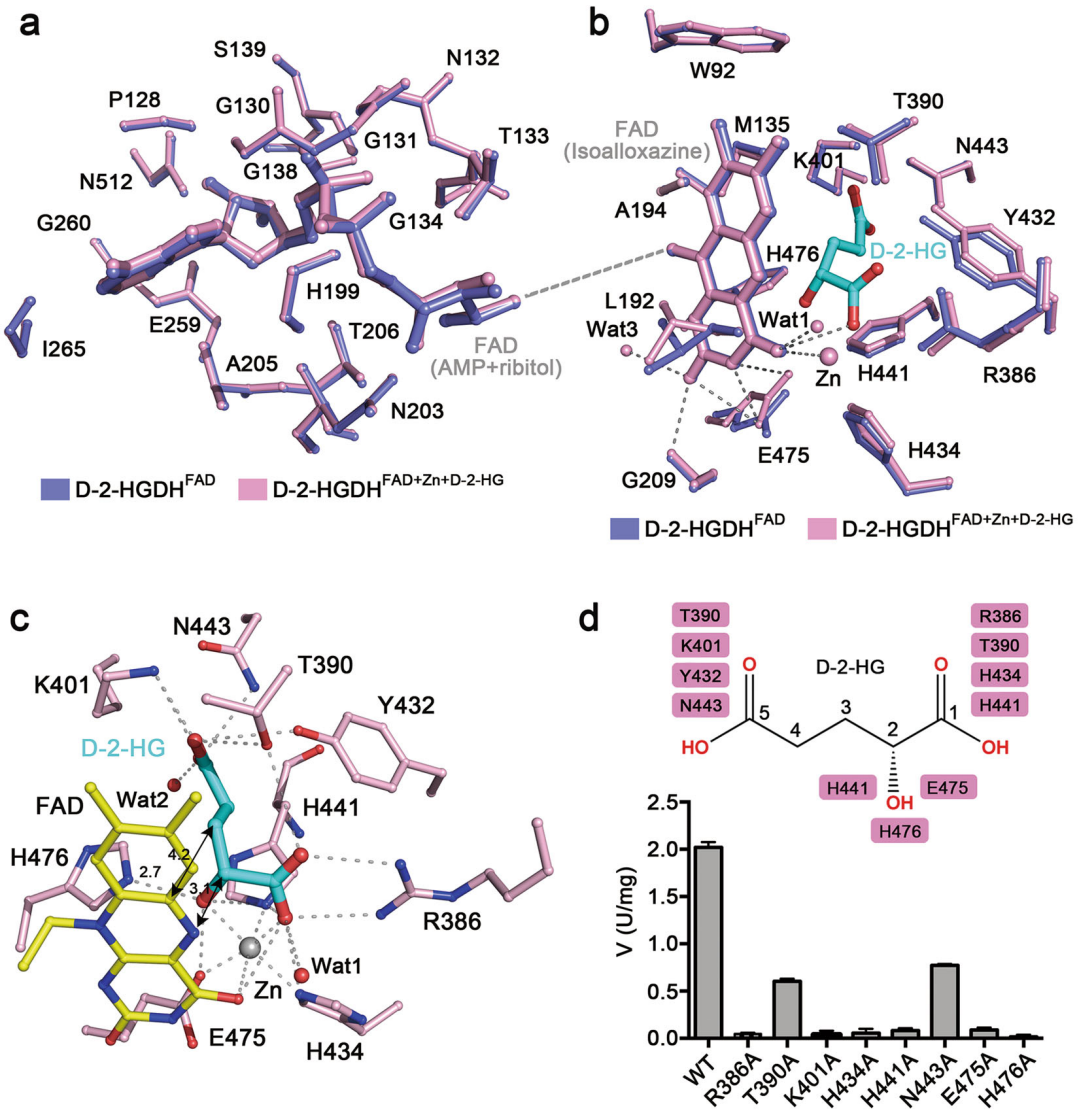
Structure of the active site of D-2-HGDH. **a** Superposition of the FAD-binding site in D-2-HGDH^FAD^ (blue) and D-2-HGDH^FAD+Zn+D-2-HG^ (pink). For clarity, only the AMP and ribitol moieties of FAD and the surrounding residues are shown with ball-and-stick models. **b** Superposition of the substrate-binding site in D-2-HGDH^FAD^ (blue) and D-2-HGDH^FAD+Zn+D-2-HG^ (pink). D-2-HG, the isoalloxazine moiety of FAD and the surrounding residues are shown with ball-and-stick models. For clarity, only the coordination bond and hydrogen bonds of the isoalloxazine moiety of FAD with Zn^2+^, D-2-HG, surrounding residues, and water molecules are indicated with dashed lines. The views of the two panels are related to each other via the linkage between the ribitol and isoalloxazine moieties of FAD as indicated by a dashed line. **c** Structure of the substrate-binding site in D-2-HGDH^FAD+Zn+D-2-HG^. The Zn^2+^ is shown with a gray sphere and water molecules with red spheres. The Zn^2+^ coordination bonds, and the hydrogen-bonding and salt-bridging interactions of D-2-HG with the isoalloxazine moiety of FAD and surrounding residues are indicated with dashed lines. The distances of a few key interactions are marked. **d** Mutational analysis of the key residues involved in the binding of D-2-HG. The schematic diagram shows the interactions of D-2-HG with the surrounding residues, and the graph shows the activities of wild-type and mutant D-2-HGDH. The values are the means ± SD of two independent determinations.

The FAD-binding site is located at the interface of the two subdomains of the FAD-binding domain and is covered by the small C-terminal domain (Fig. 1a). In all the D-2-HGDH structures, the FAD assumes an elongated conformation with the isoalloxazine ring in a distal position from the AMP moiety (Fig. 1 and Supplementary Fig. S9). The structural comparison shows that the binding of Zn^2+^ and D-2-HG at the active site does not induce notable conformational changes in the overall structure and at the FAD-binding site (Supplementary Table S2), and hence does not affect the conformation of FAD and its interactions with the protein (Fig. 2a, b). As the FAD-binding site is buried in the deep end of the active site, the AMP and ribitol moieties of FAD are solvent inaccessible and form both hydrophilic and hydrophobic interactions with many residues from the FAD-binding domain and a few residues from the C-terminal domain (Fig. 2a and Supplementary Fig. S9). The isoalloxazine moiety of FAD lies at the juncture of the FAD-binding domain and the substrate-binding domain with the *si* side facing towards the D-2-HG and Zn^2+^, and thus in addition to making interactions with a number of residues from the FAD-binding domain, it also makes interactions with the Zn^2+^, D-2-HG, and several residues from the substrate-binding domain (Fig. 2b and Supplementary Fig. S9). In particular, the O4 atom of the isoalloxazine ring is involved in the coordination of Zn^2+^ and forms two hydrogen bonds with the C1-carboxyl of D-2-HG and a water molecule (Wat1), and the O2 atom forms three hydrogen bonds with the main-chain amine of Gly209, the side chain of Glu475, and a water molecule (Wat3). Most of the residues involved in the FAD binding are highly conserved in the 2-hydroxy acid dehydrogenase subfamily (Supplementary Fig. S10), consistent with the observation that the FAD-binding domain is structurally conserved in the VAO/PCMH family members^17,31^.

### D-2-HG binding

The substrate-binding site is located in opposite to the *si* face of the isoalloxazine ring of FAD at the active site (Fig. 1). It is a positively charged pocket composed of several conserved hydrophilic residues including Arg386, Thr390, Lys401, Tyr432, His434, His441, Asn443, Glu475, and His476 (Fig. 2b). In the apo structure, the substrate-binding site is occupied by several water molecules (Supplementary Fig. S4b); in the D-2-HG-bound structure, there are a Zn^2+^ and a D-2-HG bound at the substrate-binding site (Supplementary Fig. S4c). The structural comparison shows that the binding of Zn^2+^ and D-2-HG does not induce notable conformational changes at the substrate-binding site as well except for Glu475 which adopts a different side-chain conformation to interact with the Zn^2+^ (Fig. 2b). The Zn^2+^ is coordinated by six ligands, including the side chains of His434, His441, and Glu475, the O4 atom of FAD, and the C1-carboxyl and C2-hydroxyl of D-2-HG (Fig. 2c). The lactate moiety of D-2-HG is positioned in parallel to the isoalloxazine ring of FAD and the acetate moiety is extended into the deep end of the substrate-binding pocket; both moieties are recognized by several conserved residues (Fig. 2c). Specifically, the C1-carboxyl of D-2-HG forms several hydrophilic interactions with the side chains of Arg386, Thr390, His434, and His441, two hydrogen bonds with the O4 atom of FAD and a water molecule (Wat1), and a coordination bond with the Zn^2+^. The C2-hydroxyl of D-2-HG forms hydrogen bonds with the side chains of His441, Glu475, and His476, and a coordination bond with the Zn^2+^. The C5-carboxyl of D-2-HG forms a salt bridge with the side chain of Lys401 and four hydrogen bonds with the side chains of Thr390, Tyr432, and Asn443, and a water molecule (Wat2). The residues involved in the Zn^2+^ coordination (His434, His441, and Glu475) are strictly conserved in the D-2-HGDH orthologues (Supplementary Fig. S10). The residues involved in the binding of the lactate moiety of D-2-HG (Arg386, His434, His441, Glu475, and His476) are also invariable in the orthologues. Among the residues involved in the binding of the C5-carboxyl of D-2-HG, Thr390 and Tyr432 are highly conserved, and Lys401 and Asn443 are strictly conserved in the orthologues.

To validate the functional roles of the key residues at the active site, we performed mutagenesis and enzymatic activity assay. Circular dichroism (CD) analyses of the wild-type and mutant D-2-HGDH proteins show that the mutations have no effect on the protein folding (Supplementary Fig. S11). The enzymatic activity assay results show that single mutations of the three key residues His434, His441 and Glu475 involved in the binding of Zn^2+^ and the C1-carboxyl and C2-hydroxyl of D-2-HG, led to complete loss of the activity towards D-2-HG (Fig. 2d and Supplementary Fig. S12a and Table S1). Mutations of Arg386 and His476, the other two residues involved in the binding of the C1-carboxyl and C2-hydroxyl of D-2-HG, also led to the ablation of the activity. Among the residues involved in the binding of the C5-carboxyl of D-2-HG (Thr390, Lys401, Tyr432, and Asn443), mutation of Lys401 abolished the activity; mutations of Thr390 and Asn443 substantially impaired the activity (by 2.6–3.4 folds) with a slightly effect on the *K*_m_ (1.2–2.0 folds) but a severe effect on the *k*_cat_ (2.6–3.8 folds) (Fig. 2d and Supplementary Fig. S12a and Table S1). However, the D-2-HGDH mutant containing mutation of Tyr432 could not be expressed for unknown reason(s). Taken together, our structural and biochemical data indicate that His434, His441 and Glu475 play an important role in the binding of Zn^2+^; Arg386 and Lys401 play a critical role in the binding of the C1- and C5-carboxyls of D-2-HG, respectively; and His476 plays a vital role in the binding of the C2-hydroxyl of D-2-HG.

### Substrate specificity and stereo selectivity

**Fig. 3:**
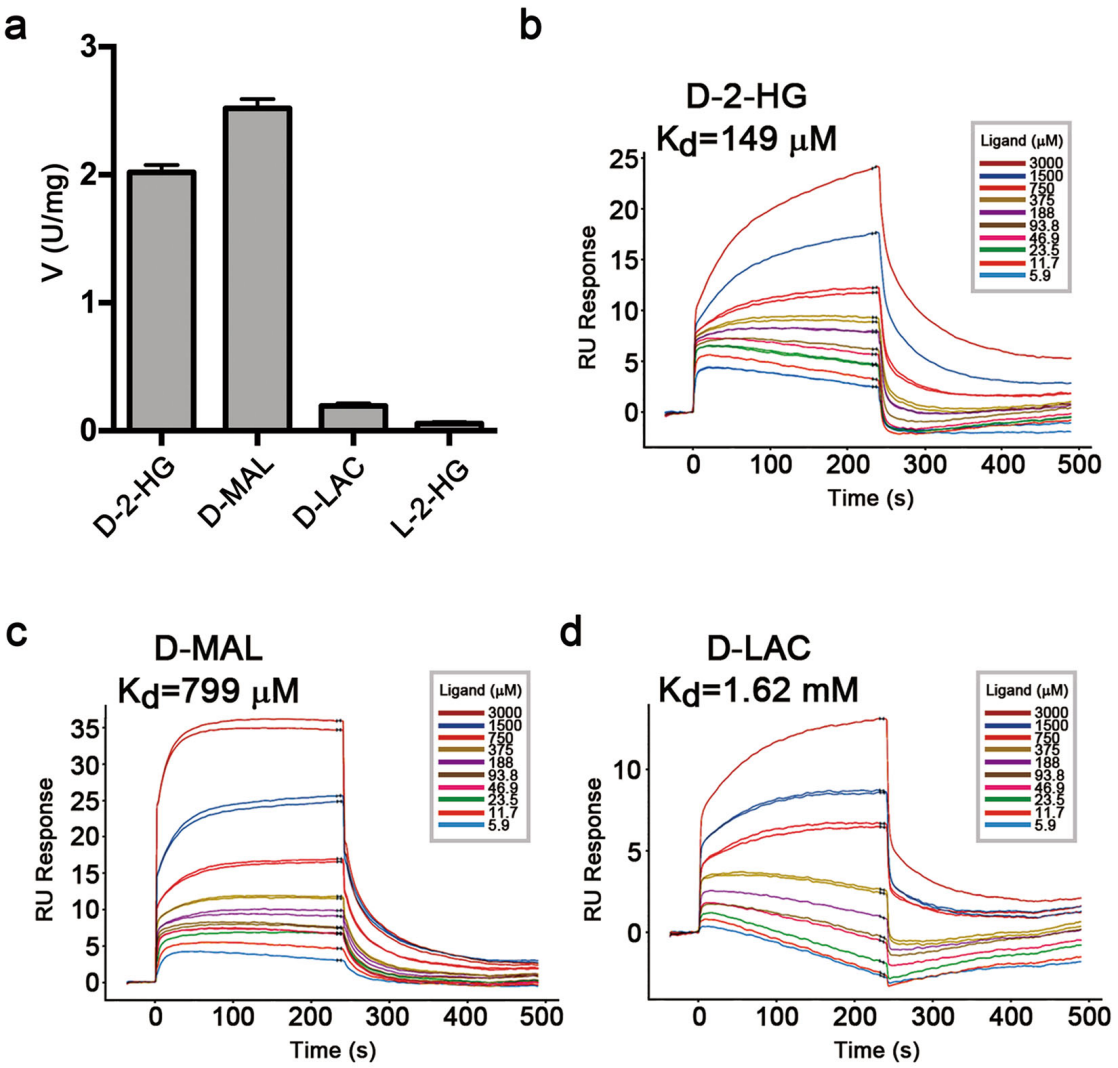
Activity and binding affinity of D-2-HGDH towards different substrates. **a** Activities of D-2-HGDH towards D-2-HG, D-MAL, and D-LAC. The values are the mean ± SD of two independent determinations. **b**–**d** SPR analysis of D-2-HGDH binding with (**b**) D-2-HG, (**c**) D-MAL, and (**d**) D-LAC.

Previous biochemical data showed that D-2-HGDH exhibits high activity towards D-2-HG, moderate activity towards D-LAC and D-MAL, but no activity towards L-2-HG^17,38,39^. Our biochemical data show that D-2-HGDH exhibits high specific activities towards both D-2-HG (2.02 ± 0.04 μmol/min/mg) and D-MAL (2.52 ± 0.05 μmol/min/mg), a very weak activity towards D-LAC (0.16 ± 0.01 μmol/min/mg), and a negligible activity towards L-2-HG (0.06 ± 0.01 μmol/min/mg) (Fig. 3a and Supplementary Fig. S12b and Table S1). Compared to D-2-HG, D-2-HGDH exhibits a slightly weaker binding (*K*_m_) with D-MAL (1.4-fold) but has a slightly higher *k*_cat_ for D-MAL (1.3-fold). Although D-2-HGDH exhibits also a slightly weaker binding (*K*_m_) with D-LAC (1.3-fold), it has a significantly low *k*_cat_ for D-LAC (13.5-fold) (Supplementary Table S1). Similar results were also observed for *P. stutzeri* D-2-HGDH^39^. Consistently, SPR analyses show that D-2-HGDH has a tighter binding with D-2-HG (*K*_d_ of 149 μM) than D-MAL (*K*_d_ of 799 μM) and D-LAC (*K*_d_ of 1.62 mM) (Fig. 3b–d), but exhibits no measurable binding with L-2-HG. Intriguingly, although the purified D-2-HGDH protein (in which the bound FAD is presumably in the oxidized form) has no measurable binding with 2-OG, the DTT treated protein (in which the bound FAD is presumably in the reduced form) exhibits a weak binding with 2-OG (*K*_d_ of 7 mM) (Supplementary Fig. S13). Similar results were also observed for VAO^40^. The differed binding affinities of 2-OG to the oxidized and reduced enzyme suggest that the re-oxidation of FAD by dioxygen during catalysis may facilitate the product release.

D-2-HG, D-MAL, and D-LAC share similar chemical structures, which all contain a D-lactate moiety but a differed moiety at the C3-position: D-2-HG has an acetate; D-MAL has a formate; and D-LAC has a hydrogen (Supplementary Fig. S2). L-2-HG is the enantiomer of D-2-HG, and 2-OG is the oxidized product of D-2-HG which has a 2-oxo at the C2 atom (Supplementary Fig. S2). To understand the molecular basis of the substrate specificity and stereo selectivity of D-2-HGDH, we determined the structures of D-2-HGDH in complexes with D-MAL, D-LAC, L-2-HG, and 2-OG. In the structures of D-2-HGDH in complexes with D-MAL and D-LAC, there is clearly defined electron density for the Zn^2+^ and D-MAL or D-LAC at the active site (Supplementary Fig. S4d, e).

**Fig. 4:**
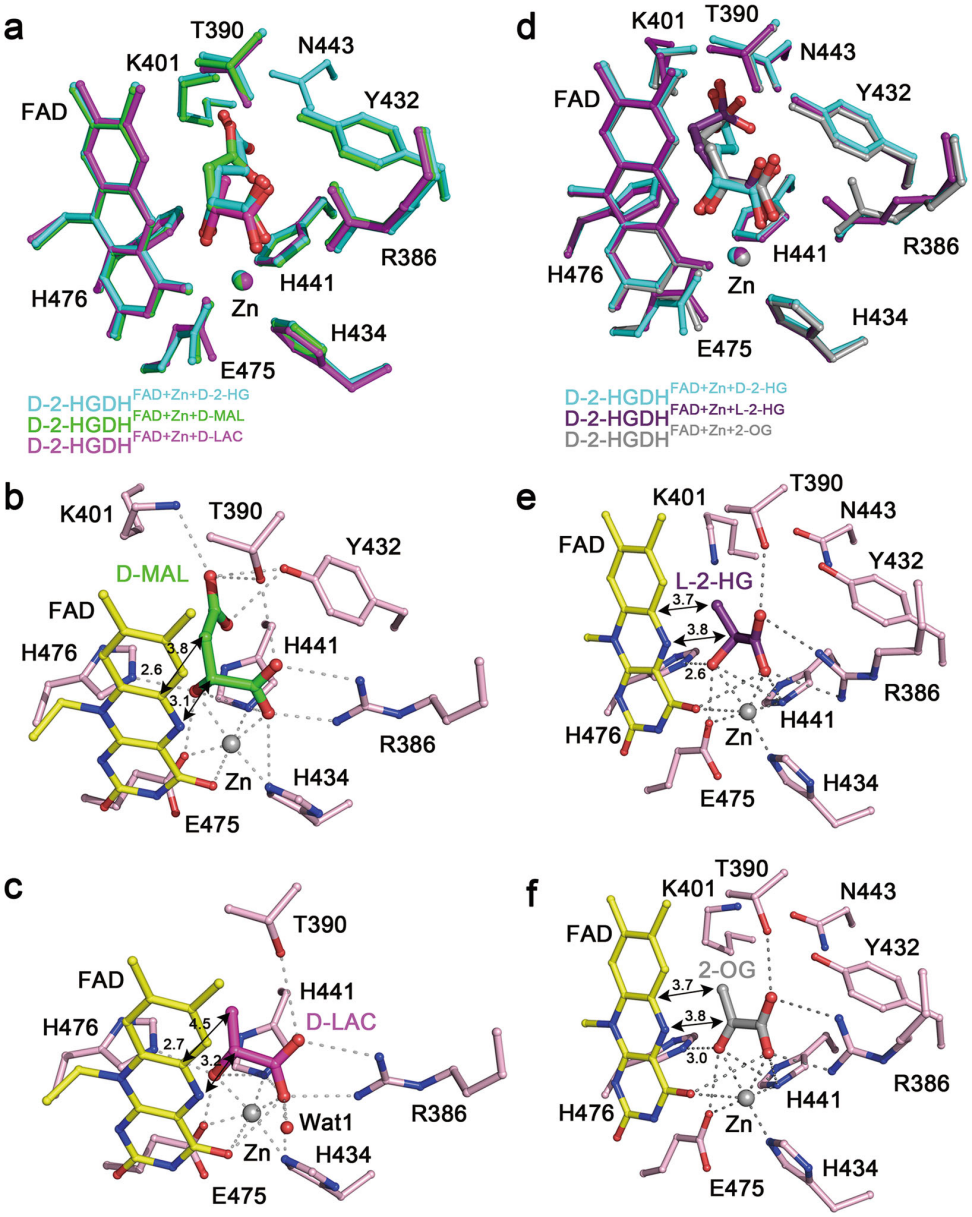
Substrate specificity and stereo selectivity of D-2-HGDH. **a** Superposition of the substrate-binding sites in D-2-HGDH^FAD+Zn+D-2-HG^ (cyan), D-2-HGDH^FAD+Zn+D-MAL^ (green), and D-2-HGDH^FAD+Zn+D-LAC^ (pink). D-2-HG/D-MAL/D-LAC, the isoalloxazine moiety of FAD and the surrounding residues are shown with ball-and-stick models. **b** Structure of the substrate-binding site in D-2-HGDH^FAD+Zn+D-MAL^. **c** Structure of the substrate-binding site in D-2-HGDH^FAD+Zn+D-LAC^. The Zn^2+^ coordination bonds, the hydrogen-bonding and salt-bridging interactions of D-MAL or D-LAC with the isoalloxazine moiety of FAD and the surrounding residues are indicated with dashed lines. The distances of a few key interactions are marked. **d** Superposition of the substrate-binding sites in D-2-HGDH^FAD+Zn+D-2-HG^ (cyan), D-2-HGDH^FAD+Zn+L-2-HG^ (violet), and D-2-HGDH^FAD+Zn+2-OG^ (gray). D-2-HG/L-2-HG/2-OG, the isoalloxazine moiety of FAD and the surrounding residues are shown with ball-and-stick models. The acetate moiety at the C3-position of L-2-HG or 2-OG is disordered and thus is modeled based on the position of the lactate moiety of L-2-HG or the pyruvate moiety of 2-OG. **e** Structure of the substrate-binding site in D-2-HGDH^FAD+Zn+L-2-HG^. The acetate moiety at the C3-position of L-2-HG is disordered and thus only the lactate moiety of L-2-HG is shown. **f** Structure of the substrate-binding site in D-2-HGDH^FAD+Zn+2-OG^. The acetate moiety at the C3-position of 2-OG is disordered and thus only the pyruvate moiety of 2-OG is shown.

Comparison of the D-2-HG-, D-MAL-, and D-LAC-bound structures reveals no notable conformational changes in the overall structure and at the active site (Fig. 4a and Supplementary Table S2). D-MAL and D-LAC bind to the same position as D-2-HG. In particular, the C1-carboxyl and C2-hydroxyl of D-MAL and D-LAC occupy almost identical positions and maintains similar interactions with the Zn^2+^ and surrounding residues as those of D-2-HG (Fig. 4a–c). As D-MAL is one carbon shorter than D-2-HG, while the C4-carboxyl group retains interactions with Thr390, Lys401, and Tyr432, it has less interactions with the protein than the C5-carboxyl of D-2-HG (Fig. 4b). This might explain why D-2-HGDH shows a higher affinity with D-2-HG than D-MAL in the SPR analysis and a slightly higher *K*_m_ value in the kinetic analysis (Fig. 3b, c and Supplementary Table S1). Compared to D-2-HG and D-MAL, D-LAC has much less interactions with the protein due to the lack of a functional group at the C3-position (Fig. 4c). These results are consistent with the biochemical data showing that D-2-HGDH has a higher binding affinity for D-2-HG than D-MAL and D-LAC (Fig. 3b–d). The structural and biochemical data together provide the molecular basis for why D-2-HGDH has broad substrate specificity towards D-2-HG, D-MAL, and D-LAC, and exhibits high activities towards D-2-HG and D-MAL but a very weak activity towards D-LAC.

In the structures of D-2-HGDH in complexes with L-2-HG and 2-OG, there is clearly defined electron density for Zn^2+^ and the lactate moiety of L-2-HG or the pyruvate moiety of 2-OG at the active site; however, the acetate moiety of both L-2-HG and 2-OG is disordered, indicating that the acetate moiety of L-2-HG and 2-OG has flexible conformation (Supplementary Fig. S4f, g). Comparison of the D-2-HG-, L-2-HG-, and 2-OG-bound structures also reveals no notable conformational changes in the overall structure and at the active site (Fig. 4d and Supplementary Table S2). In the L-2-HG-bound structure, the C1-carboxyl of L-2-HG occupies a similar position and maintains similar interactions with the Zn^2+^ and surrounding residues as that of D-2-HG (Fig. 4d, e). However, due to differed chirality of the C2 atom, to avoid steric conflicts of the C3 atom and the attached acetate moiety of L-2-HG with the isoalloxazine ring of FAD, the C2 atom is pushed away from the N5 atom of the isoalloxazine ring by 0.7 Å compared to that of D-2-HG (3.8 Å vs. 3.1 Å) and thus is not in a proper position for dehydrogenation. Concurrently, the C3 atom moves slightly closer to the C5X atom of the isoalloxazine ring by 0.5 Å (3.7 Å vs. 4.2 Å). It is conceivable that the acetate moiety of L-2-HG has to assume a different conformation from that of D-2-HG and hence makes less interactions with the protein, leading to its disordering. This is consistent with the biochemical data showing that D-2-HGDH has no measurable binding to L-2-HG. The structural and biochemical data together provide the molecular basis for the stereo selectivity of D-2-HGDH for D-2-HG against L-2-HG and explain why D-2-HGDH has no activity towards L-2-HG.

Similarly, in the 2-OG-bound structure, the C1-carboxyl and C2-oxo of 2-OG assume similar conformations and maintain similar interactions with the surrounding residues as the C1-carboxyl and C2-hydroxyl of D-2-HG (Fig. 4d, f). However, due to the coplanar geometry of the C2 atom, to avoid the steric conflicts of the C3 atom and the attached acetate moiety with the isoalloxazine ring of FAD, the C2 atom is also pushed away from the N5 atom of the isoalloxazine ring by 0.7 Å compared to that of D-2-HG (3.8 Å vs. 3.1 Å), and the C3 atom is positioned slightly closer to the C5X atom of the isoalloxazine ring by 0.5 Å (3.7 Å vs. 4.2 Å). Consequently, the acetate moiety of 2-OG has to assume a different conformation from that of D-2-HG and thus makes less interactions with the protein, leading to its disordering. This is also consistent with the biochemical data showing that D-2-HGDH in the oxidized form has no measurable binding to 2-OG and D-2-HGDH in the reduced form has weak binding to 2-OG (Supplementary Fig. S13). The structural and biochemical data together indicate that D-2-HGDH has a weak binding to 2-OG, which facilitates the release of 2-OG from the active site.

### Catalytic mechanism

The catalytic reaction mechanism has been proposed for the VAO/PCMH flavoprotein family members based on the structural and functional data^40–42^. Although these enzymes can catalyze a broad range of chemical reactions through oxidation, reduction or non-redox conversion of their substrates and may or may not utilize a metal ion to stabilize the substrate binding, one or more polar residues are always employed to function as Lewis base to deprotonate a hydroxyl of the substrate and then a hydride anion is transferred to the N5 atom of FAD to form a flavin hydroquinone anion. Our structural and biochemical studies of D-2-HGDH have identified the key residues involved in the binding of Zn^2+^, FAD, and D-2-HG, and their functional roles in the catalysis. In particular, His434, His441, and Glu475 play an important role in the binding of Zn^2+^; Arg386 and Lys401 play a critical role in the binding of the C1- and C5-carboxyls of D-2-HG, respectively; and His476 plays a vital role in the binding of the C2-hydroxyl of D-2-HG (Fig. 2c). Moreover, in the structures of D-2-HGDH in complexes with D-2-HG, D-MAL, and D-LAC, the side-chain Nε2 atom of His476 always forms a hydrogen bond with the C2-hydroxyl of D-2-HG (2.7 Å), D-MAL (2.6 Å) and D-LAC (2.7 Å), and is in a proper geometry (about 120°) to abstract the proton from the C2-hydroxyl of the substrate (Figs. 2c, 4b, c), suggesting that His476 could function as the Lewis base to deprotonate the C2-hydroxyl of the substrate and initiates the expulsion of a hydride anion from the C2 atom in the catalytic reaction. Although the side chains of His441 and Glu475 also make hydrogen-bonding interactions with the C2-hydroxyl of the substrate, they are not in proper geometry to deprotonate the C2-hydroxyl of D-2-HG; instead, they appear to play critical roles in coordination of the metal ion and stabilization of the substrate.

**Fig. 5:**
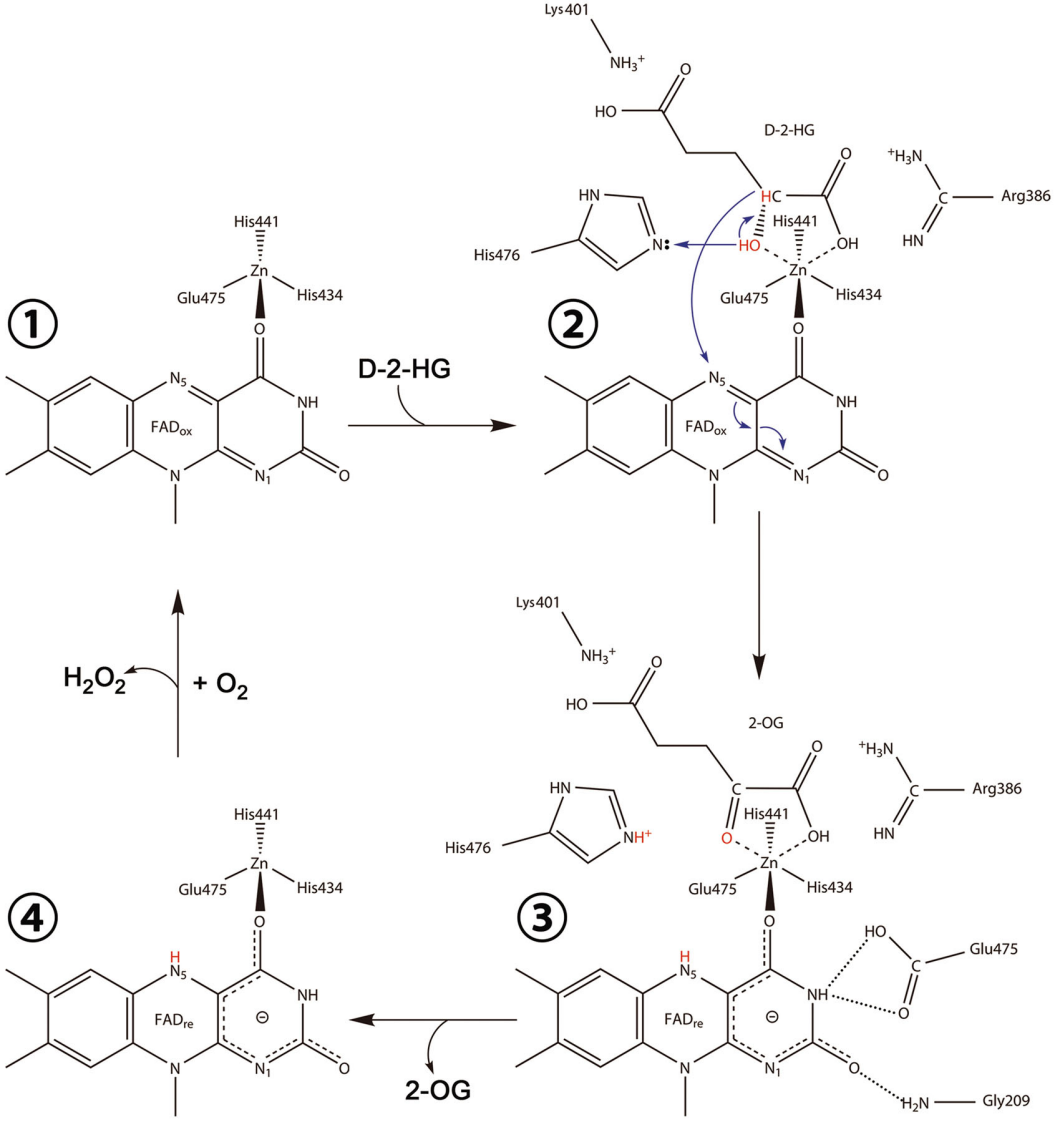
Catalytic mechanism of D-2-HGDH. The C1-carboxyl and C5-carboxyl of D-2-HG are specifically recognized by Arg386 and Lys401, respectively. The C2-hydroxyl of D-2-HG is stabilized and polarized by the Zn^2+^ and the surrounding residues. His476 acts as the Lewis base to abstract the proton from the C2-hydroxyl of D-2-HG, and concurrently a hydride anion is expulsed from the C2 atom and transferred to the N5 atom of the isoalloxazine ring of FAD, leading to the formations of the product 2-OG and a flavin hydroquinone anion. The reduced FAD is subsequently oxidized by molecule oxygen, and 2-OG is released from the active site.

Based on the structural and biochemical data, we can propose a catalytic mechanism for D-2-HGDH (Fig. 5). Firstly, D-2-HG binds to the active site with its C1-carboxyl and C5-carboxyl being recognized by Arg386 and Lys401, respectively. Secondly, the C2-hydroxyl of D-2-HG is stabilized and polarized by the Zn^2+^ and the surrounding residues, and then His476 acts as the Lewis base to abstract the proton from the C2-hydroxyl of D-2-HG. Thirdly, a hydride anion is expulsed from the C2 atom and transferred to the N5 atom of the isoalloxazine ring of FAD, leading to the formation of 2-OG and a flavin hydroquinone anion. The flavin hydroquinone anion is stabilized by the surrounding residues and Zn^2+^. The product 2-OG binds weakly to the active site due to its coplanar geometry, leading to its dissociation from the active site. Finally, the reduced FAD can be converted back into the oxidized form by molecular oxygen coupled with the generation of hydrogen peroxide.

### Functional roles of diseases associated mutations

**Fig. 6:**
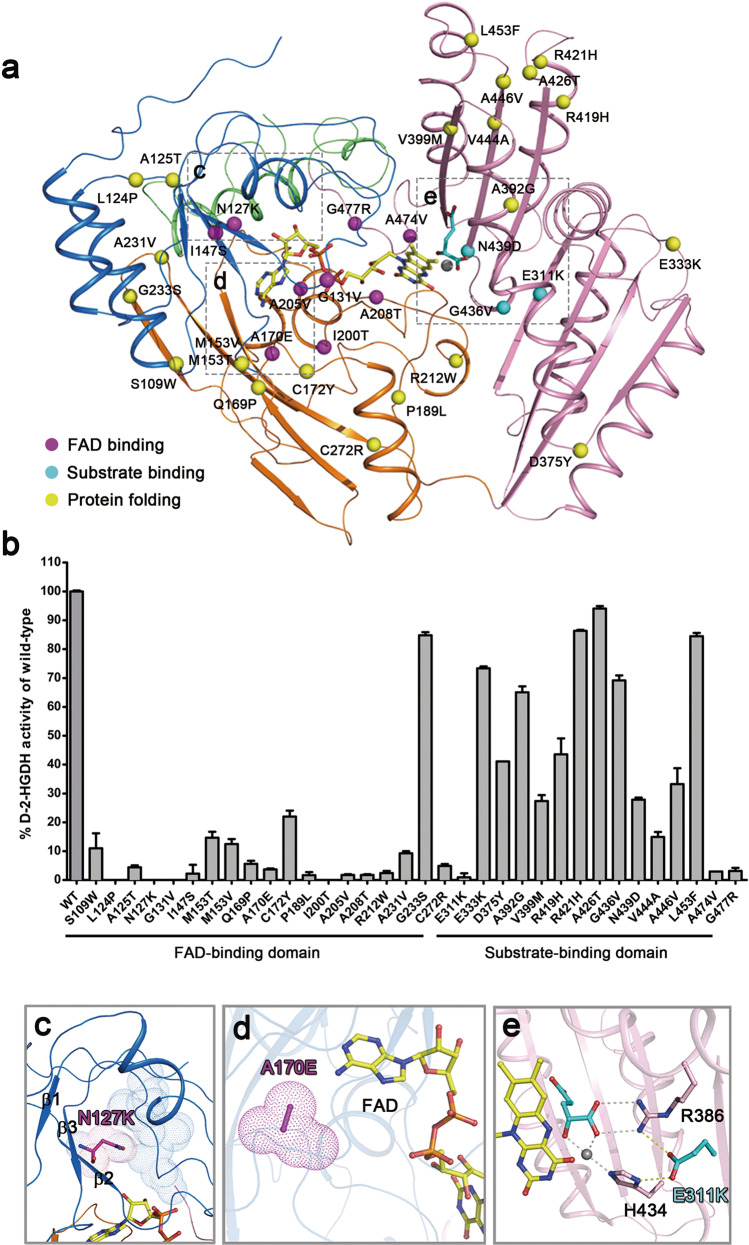
Functional and structural impacts of the diseases associated mutations of D-2-HGDH. **a** Locations of the diseases associated mutations in the structure of D-2-HGDH. Color-coding scheme of the domains is the same as in Fig. 1a. Mutations affecting the FAD binding, the substrate binding, and the protein folding and stability are shown with purple, cyan, and yellow spheres, respectively. **b** Residual activity of the diseases associated D-2-HGDH mutants, shown as % of the wild-type D-2-HGDH activity. The values are the means ± SD of two independent determinations. **c** Mutation N127K affects the conformation of the β2-β3 loop which is directly involved in the FAD binding. **d** Mutation A170E causes steric conflict with the adenine moiety of FAD. **e** Mutation E311K affects the side-chain conformations of Arg386 and His434 which are directly involved in the binding of D-2-HG and Zn^2+^.

Type I D-2-HGA is caused by deficient D-2-HGDH, and genomic analyses of type I D-2-HGA patients have identified 42 variants in the *D2HGDH* gene, of which 31 are missense variants and 11 are truncated variants^43^. In addition, genomic analyses of DLBCL patients have also identified 4 missense variants in *D2HGDH*^30^. Truncated variants are usually deemed as pathogenic, whereas the functional roles of missense variants and their pathogenicity remain elusive. A previous functional study of the missense variants associated with type I D-2-HGA showed that compared to the wild-type D-2-HGDH, 18 mutants have severely impaired activity (<6%) and 13 mutants have substantially reduced activity (17%–94%)^43^. However, as the activity assays were carried out using the cell lysates of HEK293 cells overexpressing the wild-type and mutant D-2-HGDH proteins, the measured activities seem to be inaccurate probably due to inaccurate measurement of the enzyme’s concentration caused by interference of other endogenous proteins. For instance, the activity of wild-type D-2-HGDH was determined to be 1.73 nmol/min/mg^43^, which is 3 orders of magnitude lower than the activity (2.01 μmol/min/mg) determined in this work (Fig. 3a). In addition, owing to the lack of structural information of D-2-HGDH, the molecular basis for how the mutations affect the activity is unclear. To gain further mechanistic insight into the pathogenicity of the mutations, we performed mutagenesis and enzymatic activity assays using recombinant mutant D-2-HGDH proteins, and analyzed the possible structural effects of the mutations on the binding of the cofactor and substrate and the protein folding and stability (Fig. 6 and Supplementary Table S3).

Among the type I D-2-HGA associated mutations, 17 mutations are located in the FAD-binding domain of D-2-HGDH, and 14 mutations in the substrate-binding domain (Fig. 6a and Supplementary Table S3). Among the DLBCL associated mutations, 2 mutations are located in the FAD-binding domain and the other 2 mutations in the substrate-binding domain. Compared to the wild-type D-2-HGDH, almost all the mutations in the FAD-binding domain completely ablate (<5%) or severely impair (<22%) the activity (Fig. 6b and Supplementary Table S3). Structural analyses show that several mutations are located at the FAD-binding site, including N127K, G131V, I147S, A170E, I200T, A205V, and A208T; these mutations directly affect the FAD binding and thus abolish the activity (<5%) (Fig. 6a, b and Supplementary Table S3). For examples, mutation N127K causes steric conflict with the β2-β3 loop which is directly involved in the FAD binding (Fig. 6c); and mutation A170E exhibits spatial conflict with the adenine moiety of FAD (Fig. 6d). The other mutations are not located in the vicinity of the FAD-binding site but also have severe or significant effects on the activity (4%-22%), including S109W, L124P, A125T, M153V/T, Q169P, C172Y, P189L, A231V, and C272R (Fig. 6a, b and Supplementary Table S3). These mutations appear to affect the protein folding and stability and consequently the FAD binding indirectly, leading to impairment of the activity. Mutation G233S in the FAD-binding domain is an exception that has a minor effect on the activity (85%). This mutation is located on the protein surface and appears to have no apparent impact on the function of D-2-HGDH.

In contrast, only a few mutations in the substrate-binding domain abolish the activity (<3%), including E311K, A474V, and G477R (Fig. 6a, b and Supplementary Table S3). Glu311 is located at the substrate-binding site and makes hydrophilic interactions with Arg386 and His434, both of which are directly involved in the binding of Zn^2+^ and D-2-HG; mutation E311K causes charge change at the substrate-binding site, which would disrupt these interactions and thus affect the binding of Zn^2+^ and D-2-HG (Fig. 6e). Ala474 and Gly477 are located at the FAD-binding site; mutations A474V and G477R cause steric conflicts with the FAD binding (Fig. 6a). On the other hand, most of the mutations in the substrate-binding domain have moderate or insignificant impacts on the activity and the mutants retain moderate (15%-44%) or high (65%–94%) activity (Fig. 6a, b and Supplementary Table S3). Among these mutations, G436V and N439D are located at the substrate-binding site but not directly involved in the substrate binding, and thus have insignificant or moderate impacts on the activity. The other mutations are not located in the vicinity of the substrate-binding site; they have moderate or insignificant impacts on the activity probably through affecting the protein folding and stability. Taken together, our biochemical and structural data demonstrate that the mutations in the FAD-binding domain have severe impacts and those in the substrate-binding domain have moderate or insignificant impacts on the D-2-HGDH activity.

## Discussion

Human D-2-HGDH is a member of the 2-hydroxy acid dehydrogenase subfamily of the VAO/PCMH flavoprotein family. In this work, we determined the structures of D-2-HGDH in apo form and in complexes with the substrate D-2-HG, its analogs D-MAL and D-LAC, its enantiomer L-2-HG, and the product 2-OG, and performed biochemical assays on the functional roles of the key residues involved in the substrate binding and catalytic reaction, as well as the mutations associated with type I D-2-HGA and DLBCL. Our structural and functional data not only reveal the molecular basis for the substrate specificity and catalytic mechanism of D-2-HGDH, but also shed light on the differences in function and mechanism with other VAO/PCMH flavoprotein family members.

Structural comparison shows that the structure of D-2-HGDH is very similar to the structures of representative members of the VAO/PCMH flavoprotein family, including *P. simplicissimum* VAO, *P. putida* PCMH, and *E. coli* D-LDH, despite the low sequence identity (about 10%-20%) (Supplementary Table S4 and Fig. S14a)^35,41,42^. The FAD-binding domain of these enzymes can be superimposed well albeit there are some differences among the flanking structure elements. Besides, the substrate-binding domain adopts a similar fold composed of a seven-stranded β-sheet surrounding by several α-helices. As expected, the structure of D-2-HGDH resembles mostly that of D-LDH with an RMSD of 2 Å. On the other hand, detailed structural comparisons of D-2-HGDH with D-LDH, PCMH, and VAO reveal substantial structural differences at the active site. As VAO and PCMH catalyze the oxidation of aromatic molecules, the substrate-binding sites of these two enzymes are mainly composed of hydrophobic residues, while those of D-2-HGDH and D-LDH are mainly composed of hydrophilic residues. In addition, different from the other family members who do not require metal ion for their activities^17,19^, D-2-HGDH binds a Zn^2+^ at the active site, which plays a crucial role in the binding and polarization of the substrate, and is essential for the activity. Moreover, in all known structures of the VAO/PCMH family members bound with FAD, although the AMP moiety of FAD maintains a stable conformation, the isoalloxazine ring always has few interactions with the FAD-binding domain and assumes diverse conformations (Supplementary Fig. S14b). The flexibility of the isoalloxazine ring of FAD, together with a variable substrate-binding domain, apparently allow these enzymes to form a flexible substrate-binding pocket to bind and catalyze a broad range of substrates with diverse chemical structures and properties. These differences together determine the distinct substrate specificities of different members of the VAO/PCMH family.

It is also noteworthy that in D-2-HGDH, the FAD is non-covalently bound to the enzyme, which differs from the covalent binding of FAD in many members of the VAO/PCMH family. For instances, the FAD is linked to His422 of VAO via an 8α-(*N*^3^-histidyl)-FAD bond and Tyr384 of PCMH via an 8α-(*O*-tyroxyl)-FAD bond^41,42^. The structural comparison shows that the position in D-2-HGDH equivalent to His422 of VAO or Tyr384 of PCMH is a tryptophan (Trp92) (Fig. 2b), which cannot form a covalent bond with the FAD. Intriguingly, previous biochemical studies showed that the VAO variants containing the His422 mutations which cannot form a covalent bond with the FAD exhibit significantly decreased activity compared to the wild-type enzyme^44^. Thus, it was suggested that the formation of a histidyl-FAD bond in VAO and other flavoenzymes might provide a way to increase their activities^44^. Systematic screening of substitution of Trp92 of D-2-HGDH with other residues might be able to identify possible mutation(s) which could form a covalent bond with FAD and thus the mutant(s) would have enhanced activity towards D-2-HG.

The 2-hydroxy acid dehydrogenase subfamily comprises D-2-HGDH, D-LDH, and GlcD, which all catalyze the oxidation of the 2-hydroxyl of 2-hydroxyacids containing C1-carboxyl and C2-hydroxyl^17,32,45^. As only the structure of *E. coli* D-LDH in apo form is reported so far, we performed structural and sequence comparisons of these enzymes to understand their substrate specificity and catalytic mechanism. The results show that the key residues composing the active site of D-2-HGDH, including those involved in the Zn^2+^ coordination, the binding of the C1-carboxyl and C2-hydroxyl of the substrate, and the catalytic reaction (Arg386, His434, His441, Glu475, and His476), are strictly conserved in its paralogues D-LDHs and GlcD (Supplementary Fig. S10). This provides the molecular basis for why all these enzymes use 2-hydroxyacids as substrates. On the other hand, among the residues involved in the binding of the C5-carboxyl of D-2-HG, Thr390 is replaced with a large hydrophobic residue, Lys401 and Tyr432 are replaced with residues with small side chains, and Asn443 is replaced by a hydrophobic residue in GlcD and D-LDHs, except yeast DLD2 and DLD3. In yeast DLD2 and DLD3, Lys401, Tyr432 and Asn443 of D-2-HGDH are strictly conserved, and Thr390 is substituted with a Pro, suggesting that these enzymes can bind and catalyze the oxidation of D-2-HG. Indeed, previous biochemical data showed that although yeast DLD2 and DLD3 exhibit activity towards both D-2-HG and D-LAC, the two enzymes have higher affinity and catalytic efficiency for D-2-HG than D-LAC, suggesting that they should be regarded as D-2-HGDH instead of D-LDH to a large extent^32^. Furthermore, structural comparison shows that although D-LDHs and GlcD contain the conserved hydrophilic residues to bind the C1-carboxyl and C2-hydroxyl of D-LAC and glycolate, the deep end of the substrate-binding pocket is small and hydrophobic, which could only accommodate D-LAC or glycolate but not D-2-HG and D-MAL. This explains why D-LDHs and GlcD have only activity towards D-LAC and/or glycolate but not D-2-HG and D-MAL^32,45,46^. As the key residues of D-2-HGDH involved in the binding of the C1-carboxyl and C2-hydroxyl of the substrate and the catalytic reaction are strictly conserved in D-LDHs and GlcD, it is very likely that D-LDHs and GlcD would share a similar substrate-binding mode and a common catalytic mechanism to oxidize D-LAC and glycolate.

In addition, our structural and functional data reveal the possible functional and structural roles of the mutations identified in type I D-2-HGA and DLBCL patients and shed light on the pathogenicity of these mutations. Our data show that the mutations in the FAD-binding domain have more severe impacts on the D-2-HGDH activity than those in the substrate-binding domain. Almost all the mutations located in the FAD-binding domain lead to complete ablation or severe impairment of the activity (<22%) through affecting the FAD binding directly or indirectly, including S109W, L124P, A125T, N127K, G131V, I147S, M153V, M153T, Q169P, A170E, C172Y, P189L, I200T, A205V, A208T, R212W, A231V, C272R; and these mutations are predicted to be highly pathogenic (Supplementary Table S3). On the other hand, only a few mutations located in the substrate-binding domain lead to complete loss or severe impairment of the activity, including E311K, A474V, G477R. These mutations are directly involved in the substrate or cofactor binding and thus are also highly pathogenic (Supplementary Table S3). Nevertheless, a number of mutations in the substrate-binding domain are located distantly from the substrate-binding site or FAD-binding site, and these mutations could affect the protein folding and stability and subsequently the substrate and/or cofactor binding indirectly. Some of these mutants retain moderate activity (30%-60%), including D375Y, V399M, R419H, N439D, V444A, and A446V; and these mutations are predicted to be likely pathogenic. Several other mutants retain high activity (69%-94%), including G233S, E333K, A392G, R421H, A426T, G436V, and L453F; and these mutations are predicted to be non-pathogenic (Supplementary Table S3). In addition, the impacts of the disease-associated mutations on the D-2-HGDH activity and the predicted pathogenicity of these mutations in this work are largely in agreement with the results from the previous biochemical study using cell lysates from HEK293T cells overexpressing wild-type and mutant D-2-HGDH proteins^43^, but also show some differences which could be explained by our structural data. These results might be valuable in the classification of the missense variants identified in type I D-2HGA patients and other diseases and in the determination of their pathogenicity.

Moreover, our biochemical data together with the previous biochemical data can explain why the cancer-associated IDH1 or IDH2 mutations cause drastic accumulation of D-2-HG in the tumor cells^13,22,47,48^. The IDH1 and IDH2 mutants lose the normal dehydrogenase function of converting ICT into 2-OG but gain a new reductase function of converting 2-OG into 2-HG. D-2-HGDH is the only known enzyme responsible for the conversion of D-2-HG into 2-OG to keep D-2-HG at a normal physiological level in the cells^17^. The previous biochemical data showed that the R132H/C IDH1 mutant has a high activity (350–650 μmol/min/mg) with a high *k*_cat_ (550~1000 s^−1^) to produce D-2-HG^12,47^. In contrast, D-2-HGDH has a moderate activity (2.01 μmol/min/mg) with a low *k*_cat_ (2.05 s^−1^) to consume D-2-HG, which is more than 2 orders of magnitude lower than that of the IDH1 mutant. These data suggest that in tumor cells, the ability of the IDH1 or IDH2 mutant to produce D-2-HG far outpaces the ability of D-2-HGDH to consume D-2-HG, leading to the accumulation of D-2-HG and subsequently the formation and progression of gliomas.

Since most of the disease-associated D-2-HGDH mutations lead to abolished or significantly impaired activity, it might be valuable to develop some agonists that could specifically bind to D-2-HGDH and then allosterically activate the activity of D-2-HGDH, and thus could be used for the treatment of the D-2-HGDH-deficient diseases. The structural characteristics and biochemical properties of D-2-HGDH uncovered in this work might facilitate the discovery of D-2-HGDH agonists for potential therapeutic treatment of the D-2-HGDH-deficient diseases.

## Materials and methods

### Cloning, expression, and purification

The DNA fragment encoding human D-2-HGDH was amplified by PCR from the cDNA library of human cells. The *D2HGDH* gene with the N-terminal 1–50 residues truncated (residues 51–521) was cloned into the pRSFDuet-1 vector (Novagen), which attaches an MBP tag and an HRV 3 C protease cleavage site at the N-terminus, and a His_6_ tag at the C-terminus. The N-terminal 1–50 residues of D-2-HGDH are highly variable among different species and the truncated protein is more stable than the full-length protein. The plasmid was transformed into *E. coli* BL21 (DE3) CodonPlus strain (Tiangen), and the transformed cells were grown in LB medium containing 0.05 mg/ml kanamycin at 37 °C to OD_600_ of 0.8 and then induces with 0.2 mM IPTG at 16 °C overnight. The bacterial cells were harvested by centrifugation and lysed by sonication in a lysis buffer (25 mM HEPES, pH 7.5, 200 mM NaCl, 10% glycerol, and 1 mM PMSF). The target protein was purified by affinity chromatography using an Amylose resin (New England BioLabs), and the elution was incubated with the HRV 3 C protease to remove the MBP tag. The mixture was further purified by affinity chromatography using a Ni-NTA column (Qiagen) and then gel filtration chromatography using a Superdex 200 10/300 column (GE Healthcare). Constructs of the D-2-HGDH mutants containing point mutations were generated using the QuikChange^®^ Site-Directed Mutagenesis kit (Stratagene) and verified by sequencing. Expression and purification of the mutants were the same as the wild-type protein. The purified proteins were of high purity as analyzed by SDS-PAGE, and stored in a storage buffer (25 mM HEPES, pH 7.5, 200 mM NaCl, and 5% glycerol) for structural and biochemical studies.

### Crystallization, data collection, and structure determination

Crystallization was performed using the hanging drop vapor diffusion method at 16 °C by mixing equal volume of the protein solution (10 mg/ml) and reservoir solution. Crystals of D-2-HGDH in apo form were grown in drops containing the reservoir solution of 0.1 M BIS-TRIS (pH 6.5) and 25% (w/v) PEG 3,350. Crystals of D-2-HGDH in complexes with D-2-HG, D-MAL, D-LAC, L-2-HG, and 2-OG were obtained by adding the ligand (40 mM) and ZnCl_2_ (50 mM) to drops containing the apo D-2-HGDH crystals and then soaking for 10 hours before crystal harvest. Crystals were cryoprotected using the reservoir solution supplemented with 30% glycerol and then flash-cooled in liquid N_2_. Diffraction data were collected at 100 K at beamlines of Shanghai Synchrotron Radiation Facility and National Facility for Protein Science in Shanghai, and processed with HKL3000^49^. Statistics of the diffraction data are summarized in Table 1.

The apo D-2-HGDH structure was solved by the molecular replacement (MR) method implemented in Phenix^50^ using the structure of a putative dehydrogenase RPA1076 from *Rhodopseudomonas palustris* (PDB code: 3PM9) as the search model. The structures of D-2-HGDH in complexes with D-2-HG, D-MAL, D-LAC, L-2-HG, and 2-OG were solved by the MR method using the apo structure as the search model. Model building was performed manually using Coot^51^ and structure refinement was carried out using Phenix^50^ and Refmac5^52^. Structural analysis was carried out using programs in the CCP4 suite^53^. Structural figures were prepared using PyMOL^54^. Statistics of the structure refinement and the quality of final structure models are also summarized in Table 1.

### Enzymatic activity assay

The activity of D-2-HGDH was determined by monitoring the reduction of 2,6-dichlorophenolindophenol (DCIP) spectrophotometrically at 600 nm using a Synergy Neo2 Hybrid Multi-Mode Reader (BioTek Instruments) based on a modified method described previously^2,55^. The standard reaction solution (100 μl) consisted of 50 mM HEPES (pH 7.5), 0.3 μM enzyme, 0.6 μM ZnCl_2_, 200 μM phenazinemethosulfate, 200 μM DCIP, and varied concentrations of the ligand (D-2-HG, D-MAL, D-LAC, and L-2-HG) incubated at 37 °C, using 96-well 1/2 area black plates. The reaction was initiated by the addition of the ligand. The activity is defined as the moles of DCIP reduced per min per milligram of enzyme (μmol/min/mg). One unit (U) of the activity is defined as 1 μmol of DCIP reduced per min. The specific activities of the wild-type D-2-HGDH and mutants were determined at the standard conditions with a fixed substrate concentration (1 mM). The kinetic data were measured with varied concentrations of the ligand (0–1 mM)_._ The kinetic parameters (*V*_max_, *K*_m_, and *k*_cat_) were obtained by fitting the kinetic data into the Michaelis-Menten equation “*V* = *V*_max_*[S]/(*K*_m_ + [*S*])” using program Graphpad Prism (Graphpad Software). All experiments were performed in duplicates and the values were the averages of the measurements with the standard errors.

### SPR analysis

The SPR analysis was performed using a Biacore 8 K instrument at 25 °C. Prior to analysis, the D-2-HGDH protein was exchanged to the running buffer containing 25 mM HEPES (pH 7.5) and 200 mM NaCl via gel filtration. The protein was covalently immobilized onto the sensor CM5 chip (GE Healthcare) in 10 mM sodium acetate (pH 5.5) following standard amine-coupling procedure. The analytes were used to flow over the chip surface with the response units measured. The binding kinetics was analyzed with the Biacore Insight Evaluation Software using the 1:1 binding model. The binding of D-2-HG, D-MAL, or D-LAC with D-2-HGDH could be measured reliably, but that of L-2-HG and 2-OG could not.

### CD analysis

The CD analysis was performed to examine the protein folding. The protein samples were diluted to 0.3–0.4 mg/ml with 50 mM potassium phosphate pH 7.5. The spectra were obtained using a Chirascan v100 spectrometer (AppliedPhotophysics) from 180 to 260 nm with 0.5 s time-per-point at 25 °C. The spectra data were processed using the CDNN (Circular Dichroism analysis using Neural Networks) software.

## Supplementary information

Supplementary Information

## Data Availability

Atomic coordinates and structure factors of the D-2-HGDH^FAD^, D-2-HGDH^FAD+Zn+D-2-HG^, D-2-HGDH^FAD+Zn+D-MAL^, D-2-HGDH^FAD+Zn+D-LAC^, D-2-HGDH^FAD+Zn+2-OG^, and D-2-HGDH^FAD+Zn+L-2-HG^ structures have been deposited with the Protein Data Bank under accession codes 6LPN, 6LPP, 6LPQ, 6LPT, 6LPX, and 6LPU, respectively.

## Supplementary information

**Supplementary Information** accompanies the paper at (10.1038/s41421-020-00227-0).
