# Supplementary material for: Structure, substrate specificity, and catalytic mechanism of human D-2-HGDH and insights into pathogenicity of disease-associated mutations
Source: Cell Discov. 2021 Jan 12;7:3. doi: 10.1038/s41421-020-00227-0 (PMC7801405; doi:10.1038/s41421-020-00227-0)
Supplement: Supplementary file 1 — Supplementary Information [file 41421_2020_227_MOESM1_ESM.pdf]

Supplemental information

Structure, substrate specificity, and catalytic mechanism of human D-2-HGDH and insights into pathogenicity of disease-associated mutations

Jun Yang[1,$], Hanwen Zhu[1,$], Tianlong Zhang[1], and Jianping Ding[1,2,✉]

[1] State Key Laboratory of Molecular Biology, Shanghai Institute of Biochemistry and Cell Biology, Center for Excellence in Molecular Cell Science, University of Chinese Academy of Sciences, Chinese Academy of Sciences, 320 Yue-Yang Road, Shanghai 200031, China

[2] School of Life Science, Hangzhou Institute for Advanced Study, University of Chinese Academy of Sciences, 1 Xiangshan Road, Hangzhou 310024, China

[$] These authors contributed equally: Jun Yang, Hanwen Zhu.

[✉] To whom correspondence should be addressed. E-mail: jpding@sibcb.ac.cn

Supplementary Fig. S1. Purification of human D-2-HGDH. a) Size-exclusion chromatography (SEC) analysis of D-2-HGDH. The purified D-2-HGDH shows an elution peak at about 15.3 ml corresponding to an apparent molecular weight of 50 kDa, which is in agreement with the theoretical molecular mass of D-2-HGDH (51 kDa). A Superdex 200 10/300 column (GE Healthcare) was used for gel filtration chromatography. **b)** SDS-PAGE analysis of the purified D-2-HGDH. The elution peak fraction of D-2-HGDH from the gel filtration chromatography was analyzed by SDS-PAGE and stained by Coomassie blue.

Supplementary Fig. S2. Chemical structures of different ligands. The atom positions of the ligands are indicated. D-2-HG, D-MAL, and D-LAC share similar chemical structures, all of which contain a D-lactate moiety consisting of a C1-carboxyl and a C2-hydroxyl and a chiral C2 atom with the D-configuration. L-2-HG is the enantiomer of D-2-HG, in which the chiral C2 atom has the L-configuration. 2-OG is the oxidized product of D-2-HG, which has a 2-oxo at the coplanar C2 atom.

Supplementary Fig. S3. Structural comparison of the two D-2-HGDH molecules in the asymmetric unit of the D-2-HGDH$^{FAD+Zn+D\text{-}2\text{-}HG}$ structure. a) Structures of the two D-2-HGDH molecules (A and B) in the asymmetric unit, which are colored in gray and pink, respectively. **b)** Superposition of the two D-2-HGDH molecules yields a root mean square deviation (RMSD) of 0.35 Å for 466 Cα atoms. The Zn^{2+} is shown with a sphere, and the FAD and D-2-HG are shown with ball-and-stick models.

Supplementary Fig. S4. Representative simulated annealing composite omit maps of the active site of D-2-HGDH in apo form and in complexes with the ligands at 1.0 σ contour level. a) The FAD in the apo D-2-HGDH structure. **b)** The active site in the apo D-2-HGDH structure. **c)** The active site in the D-2-HGDH$^{FAD+Zn+D\text{-}2\text{-}HG}$ structure. **b)** The active site in the D-2-HGDH$^{FAD+Zn+D\text{-}MAL}$ structure. **e)** The active site in the D-

2-HGDH$^{FAD+Zn+D\text{-}LAC}$ structure. **f)** The active site in the D-2-HGDH$^{FAD+Zn+L\text{-}2\text{-}HG}$ structure. **g)** The active site in the D-2-HGDH$^{FAD+Zn+2\text{-}OG}$ structure. Electron density maps are contoured at 1.0 σ. The ligands and surrounding residues are shown with stick models. The Zn^{2+} and water molecules are shown with gray and red spheres, respectively.

Supplementary Fig. S5. Representative simulated annealing composite omit maps of the active site of D-2-HGDH in apo form and in complexes with the ligands at 3.0 σ contour level. a) The active site in the apo D-2-HGDH structure. **b)** The active site in the D-2-HGDH$^{FAD+Zn+D\text{-}2\text{-}HG}$ structure. **c)** The active site in the D-2-HGDH$^{FAD+Zn+D\text{-}MAL}$ structure. **d)** The active site in the D-2-HGDH$^{FAD+Zn+D\text{-}LAC}$ structure. **e)** The active site in the D-2-HGDH$^{FAD+Zn+L\text{-}2\text{-}HG}$ structure. **f)** The active site in the D-2-HGDH$^{FAD+Zn+2\text{-}OG}$ structure. Electron density maps are contoured at 3.0 σ. The ligands and surrounding residues are shown with stick models. The Zn^{2+} and water molecules are shown with gray and red spheres, respectively.

Supplementary Fig. S6. Bond lengths for Zn^{2+} in different ligand-bound D-2-HGDH structures. a) The Zn^{2+}-binding site in the D-2-HGDH$^{FAD+Zn+D\text{-}2\text{-}HG}$ structure. **b)** The Zn^{2+}-binding site in the D-2-HGDH$^{FAD+Zn+D\text{-}MAL}$ structure. **c)** The Zn^{2+}-binding site in the D-2-HGDH$^{FAD+Zn+D\text{-}LAC}$ structure. **d)** The Zn^{2+}-binding site in the D-2-HGDH$^{FAD+Zn+L\text{-}2\text{-}HG}$ structure. **e)** The Zn^{2+}-binding site in the D-2-HGDH$^{FAD+Zn+2\text{-}OG}$ structure. The coordination bonds are shown as gray dashed lines with the bond length values indicated.

Supplementary Fig. S7. Topology diagram of D-2-HGDH. Three β-sheets are highlighted in shaded gray boxes. The FAD-binding subdomains a and b, the substrate-binding domain, and the small C-terminal domain are colored in marine, orange, pink, and green, respectively.

Supplementary Fig. S8. Comparison of the proposed substrate-loading channel of D-2-HGDH with the "ins" and "outs" of VAO. a) Overview of the identified paths in VAO. The VAO dimer is shown as surface representation with the two subunits colored in light-green and gray, respectively (PDB code: 2VAO)[1,2]. For clarity, only the four paths of one subunit are indicated. **b)** Overview of the substrate-loading channel of D-2-HGDH in two different orientations. The two D-2-HGDH molecules in the asymmetric unit of the D-2-HGDH$^{FAD+Zn+D\text{-}2\text{-}HG}$ structure are shown as surface representation. For clarity, only the subdomains of one D-2-HGDH molecule are colored the same as in Fig. 1c and the other molecule is colored in gray. The proposed substrate-loading channel in D-2-HGDH is structurally related to the path for the co-ligands dioxygen and hydrogen peroxide in VAO.

Supplementary Fig. S9. A schematic diagram showing the interactions of FAD with D-2-HGDH. The AMP, ribitol, and isoalloxazine moieties of FAD are indicated. The atom positions of the isoalloxazine ring are labeled. The color-coding scheme of the residues is the same as Figure 1A. The residues involved in the hydrophilic and hydrophobic interactions with the FAD are indicated with solid and dashed rectangles, respectively.

Supplementary Fig. S10. Sequence alignment of D-2-HGDH orthologues and its paralogues D-LDHs and GlcD. The sequence number and secondary structures of human D-2-HGDH are placed on the top of the alignment. Strictly conserved residues are highlighted in shaded red boxes and conserved residues in open red boxes. Abbreviations used: hs, *Homo sapiens*; dr, *Danio rerio*; dm, *Drosophila melanogaster*; rn, *Rattus norvegicus*; gg, *Gallus gallus*; xl, *Xenopus laevis*; at, *Arabidopsis thaliana*; ps, *Pseudomonas stutzeri*; rp, *Rhodopseudomonas palustris*; sc, *Saccharomyces cerevisiae*. The key residues of human D-2-HGDH involved in the Zn^{2+} coordination, the substrate binding, and the FAD binding are indicated with purple, cyan, and orange

balls, respectively.

Supplementary Fig. S11. Circular dichroism analysis of wild-type and mutant D-2-HGDH proteins containing mutations of the key residues at the active site. The protein samples were diluted to 0.3-0.4 mg/ml with 50 mM potassium phosphate (pH 7.5). The spectra were obtained using a Chirascan v100 spectrometer (AppliedPhotophysics) from 180 to 260 nm with 0.5 s time-per-point at 25 °C. The spectra data were processed using the CDNN (Circular Dichroism analysis using Neural Networks) software.

Supplementary Fig. S12. Saturation curves of wild-type and mutant D-2-HGDH. a) Saturation curves of wild-type and mutant D-2-HGDH towards D-2-HG. **b)** Saturation curves of wild-type D-2-HGDH towards different ligands. The activities of wild-type and mutant D-2-HGDH were measured at the standard conditions (50 mM HEPES, pH 7.5, 0.3 μM enzyme, 0.6 μM $ZnCl_2$, 200 μM phenazinemethosulfate, 200 μM DCIP) with varied concentrations of D-2-HG (0-1.0 mM). Data points shown are the means of two independent determinations. The error bars represent the standard deviations.

Supplementary Fig. S13. SPR analysis of the reduced D-2-HGDH binding with 2-OG. Prior to SPR analysis, the purified D-2-HGDH protein was incubated with 10 mM DTT for four hours to reduce the bound FAD into the reduced form.

Supplementary Fig. S14. Structural comparison of human D-2-HGDH with representative members of the VAO/PCMH family. a) Overall structures of human D-2-HGDH in complex with the substrate D-2-HG, *Escherichia coli* D-LDH^{FAD} (PDB code: 1F0X), *Penicillium simplicissimum* VAO in complex with an inhibitor *p*-cresol (PDB code: 1AHU), and *Pseudomonas putida* PCMH in complex with the substrate *p*-

cresol (PDB code: 1DIQ). The FAD-binding domain of the proteins is colored in gray, and the substrate-binding domain and the C-terminal domain are colored differently. **b)** Comparison of the conformation of FAD in the structures of different VAO/PCMH family members. In the structures of different VAO/PCMH family members bound with FAD, the AMP moiety of FAD maintains a stable conformation, but the isoalloxazine ring always has few interactions with the protein and can assume diverse conformations.

Supplementary Fig. S1

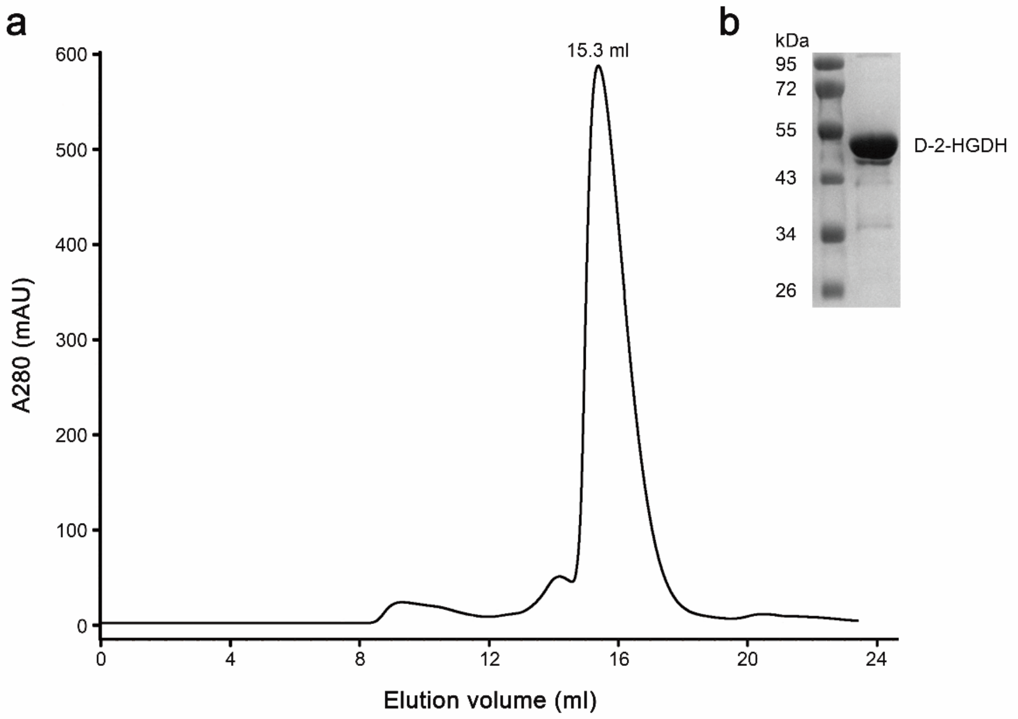

Supplementary Fig. S2

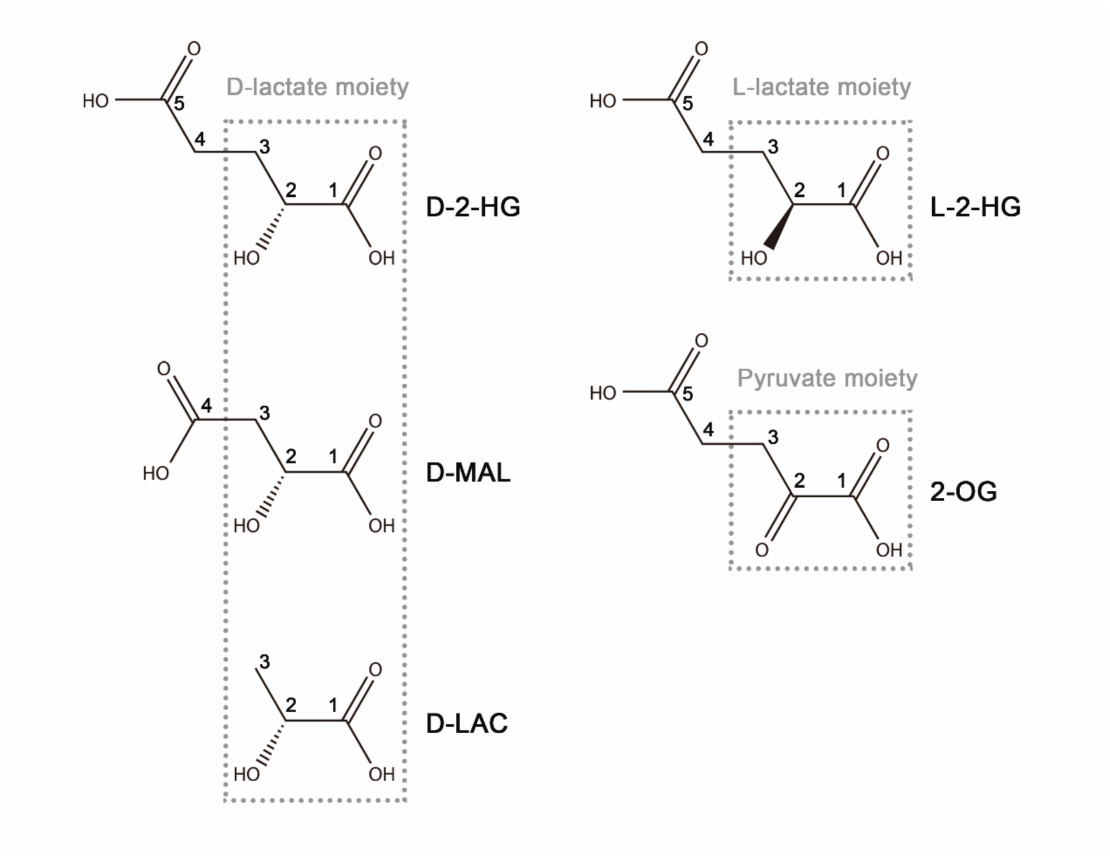

Supplementary Fig. S3

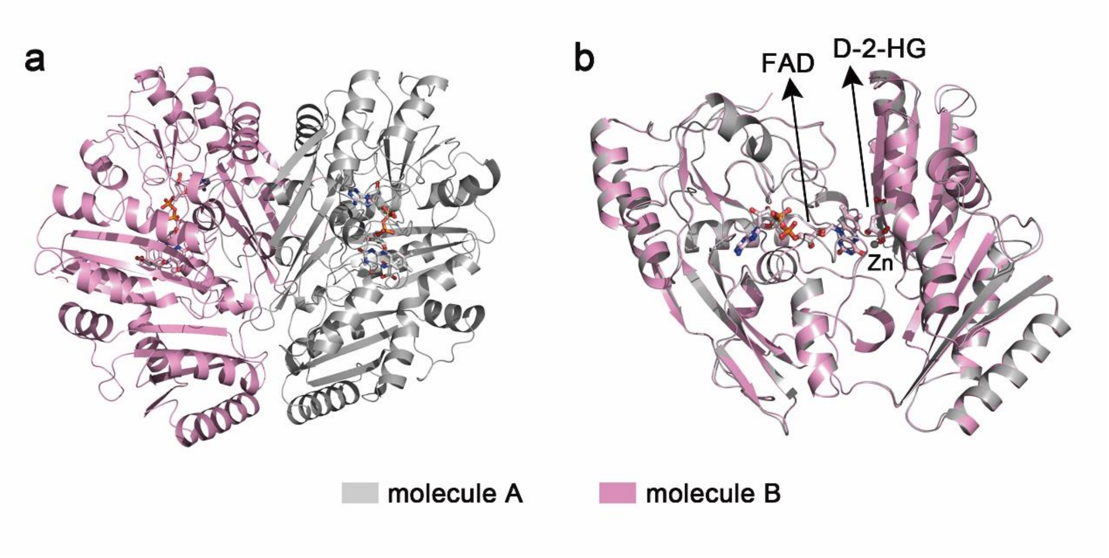

Supplementary Fig. S4

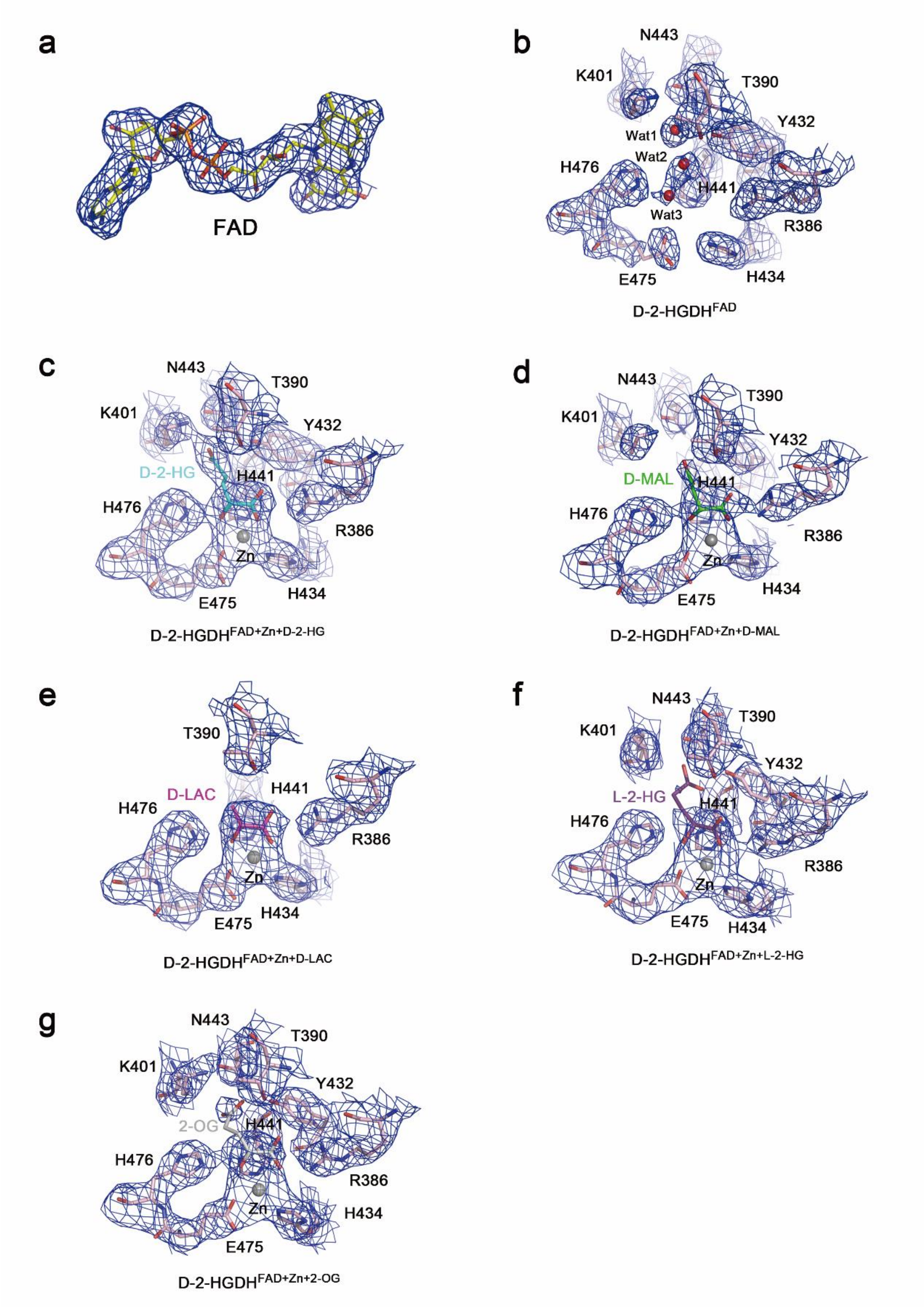

Supplementary Fig. S5

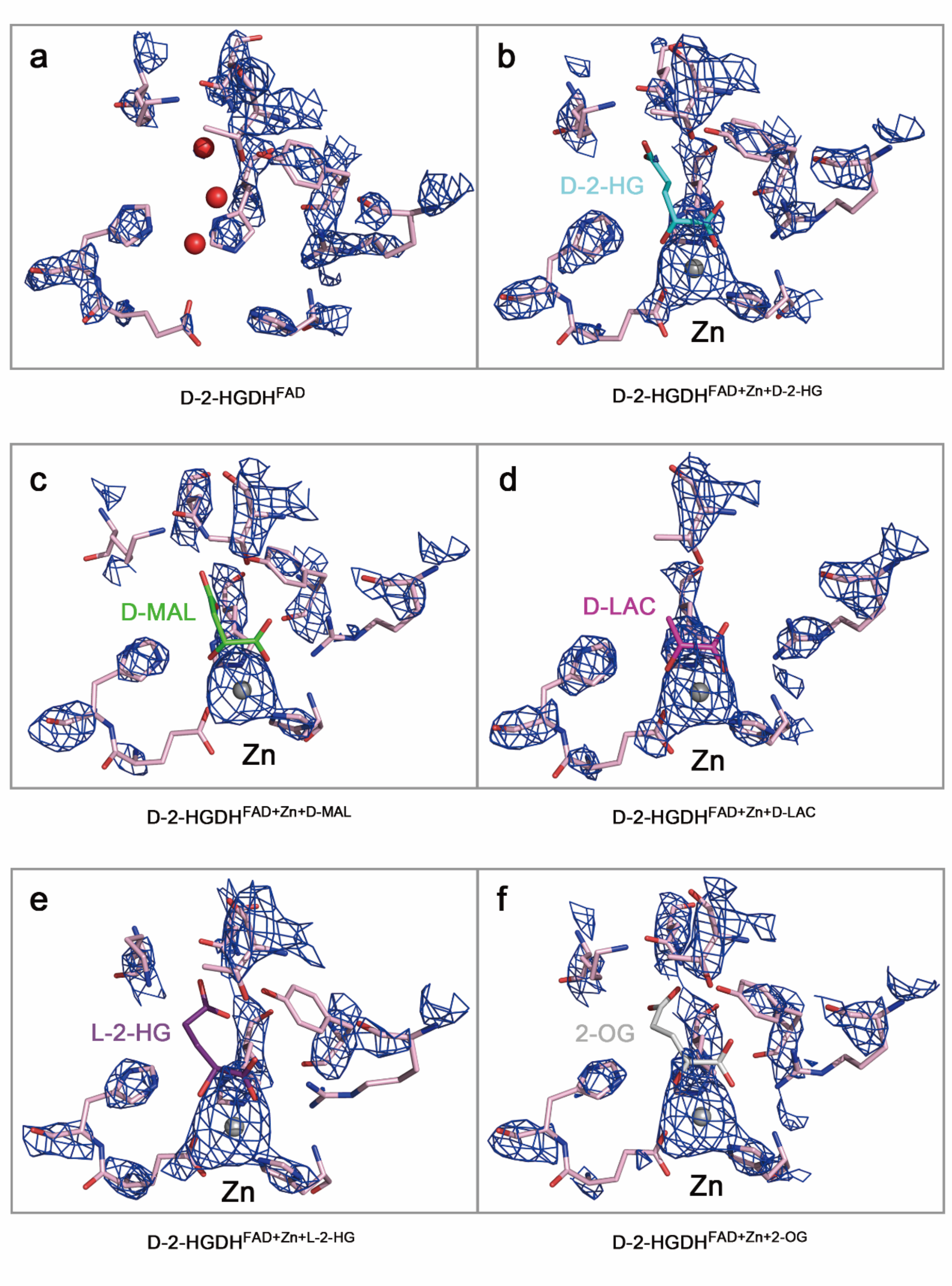

Supplementary Fig. S6

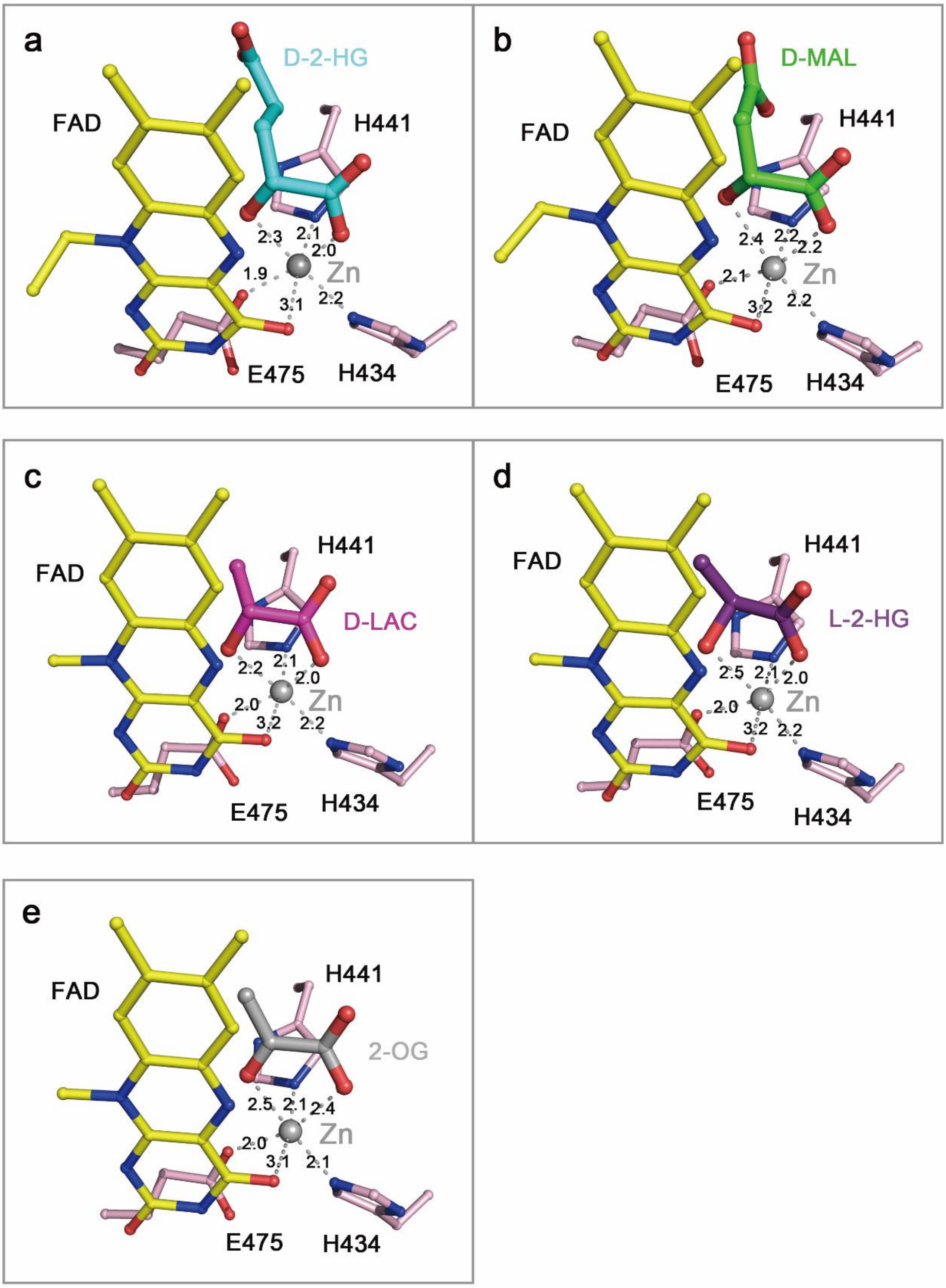

Supplementary Fig. S7

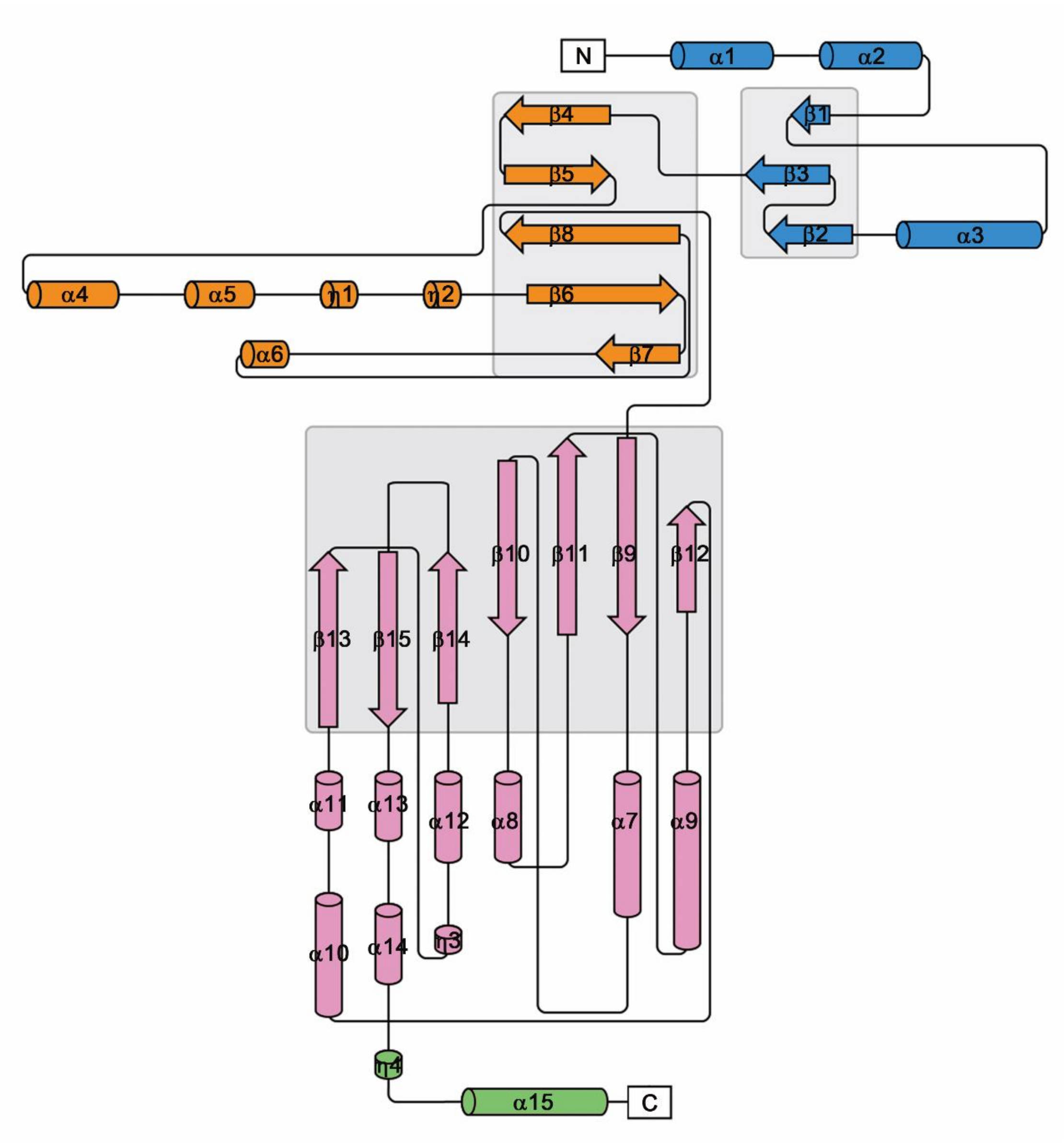

Supplementary Fig. S8

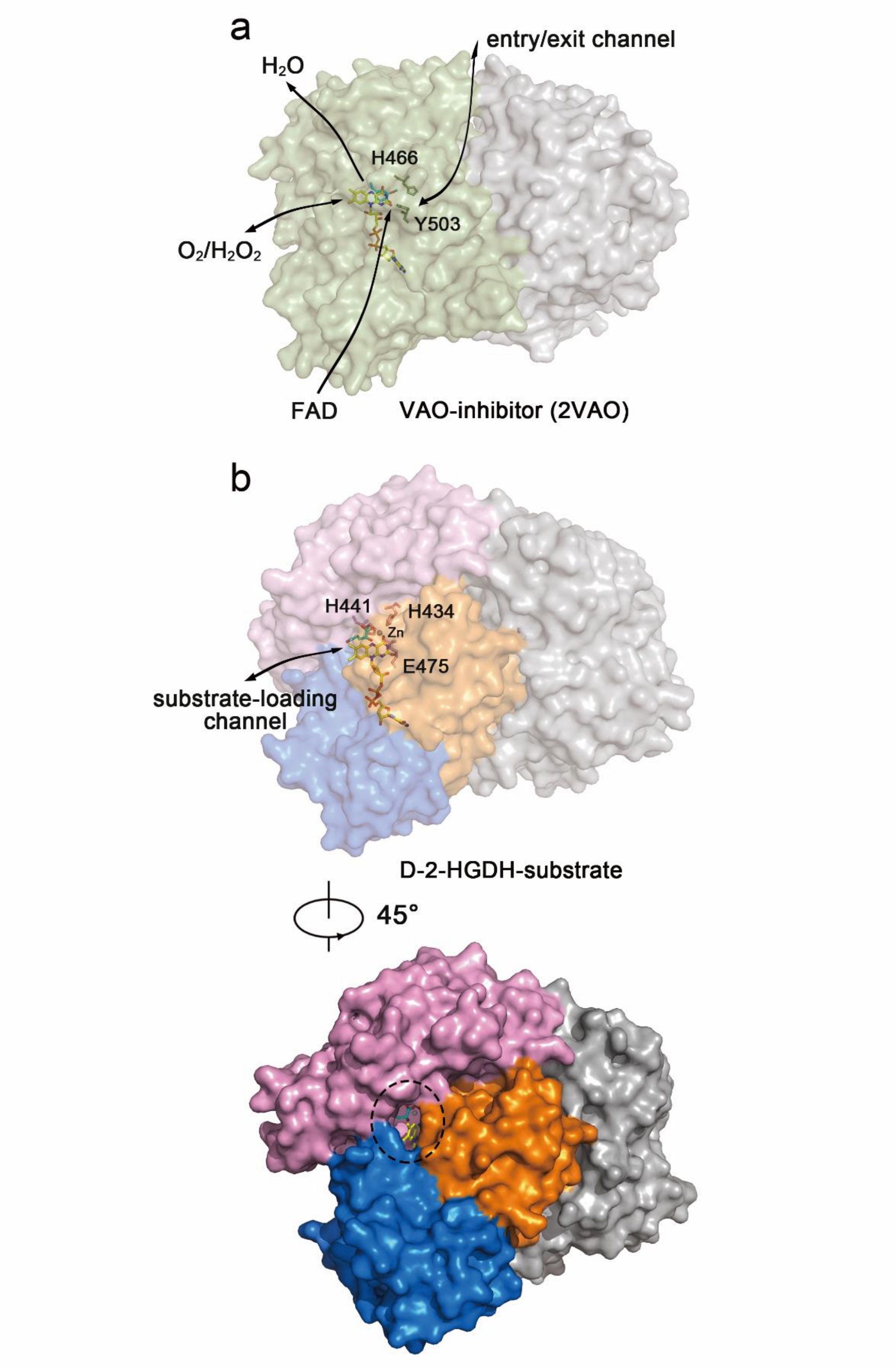

Supplementary Fig. S9

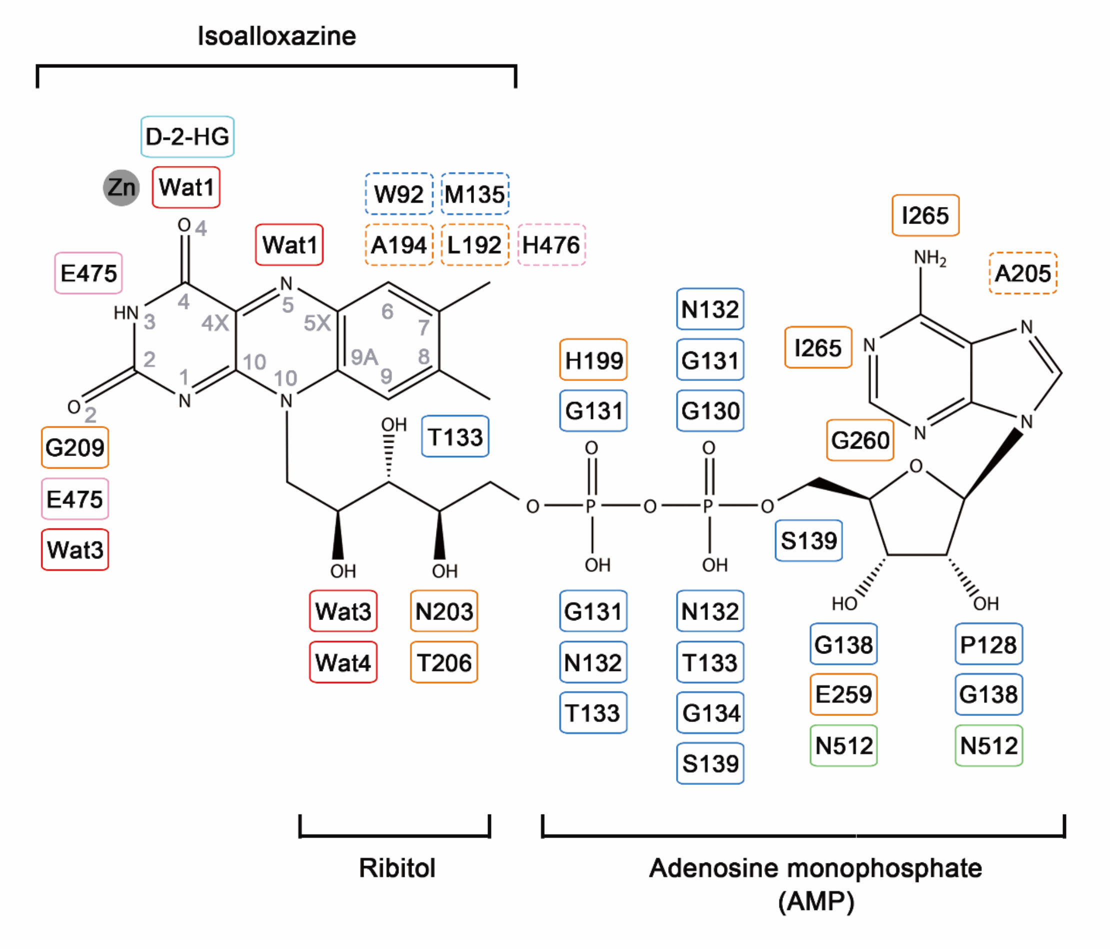

Supplementary Fig. S10

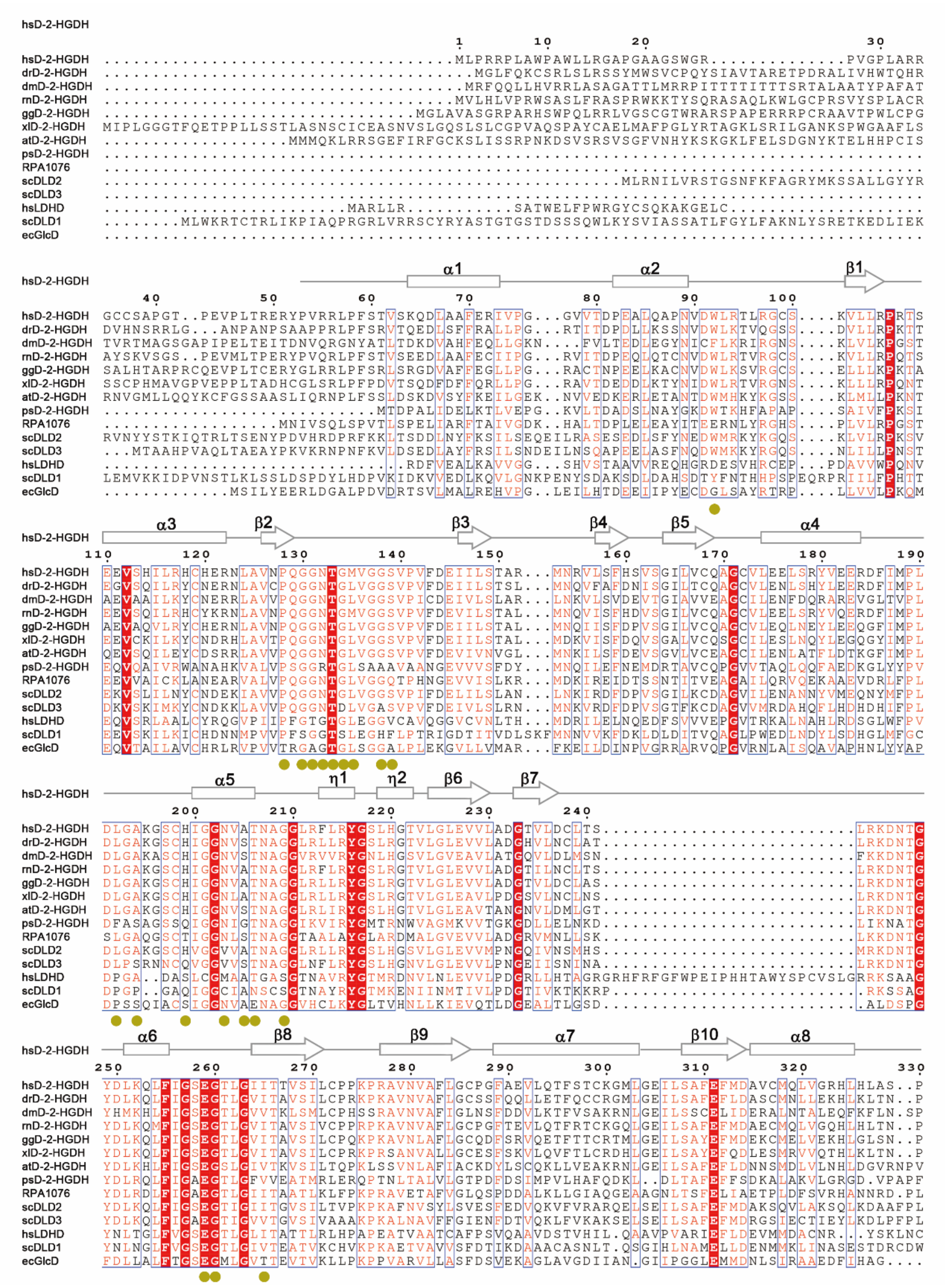

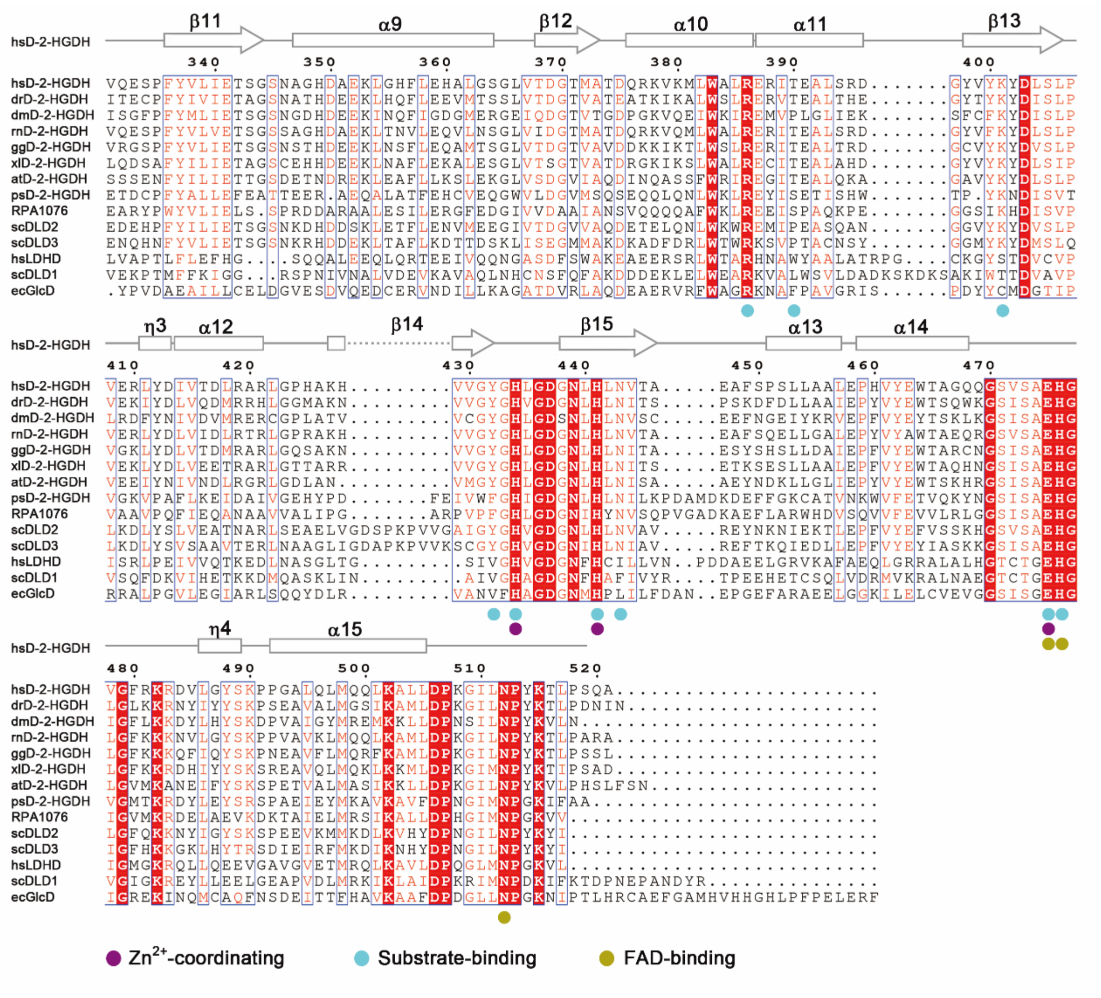
hsD-2-HGDH
β11
α9
β12
α10
α11
β13
η3
α12
β14
β15
α13
α14
η4
α15
hsD-2-HGDH
drD-2-HGDH
dmD-2-HGDH
rnD-2-HGDH
ggD-2-HGDH
xlD-2-HGDH
atD-2-HGDH
psD-2-HGDH
RPA1076
scDLD2
scDLD3
hsLDHD
scDLD1
ecGlcD
Zn2+-coordinating
Substrate-binding
FAD-binding

Supplementary Fig. S11

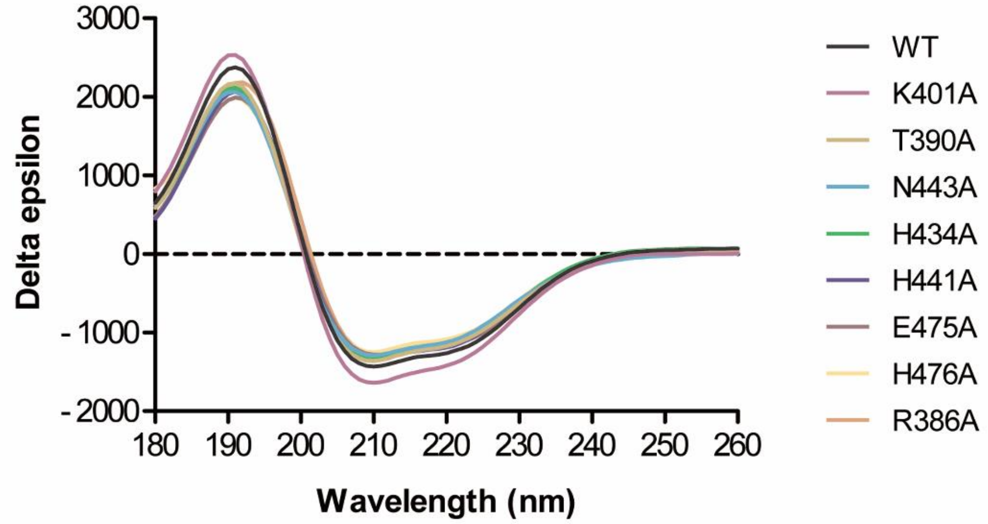

	Helix	Antiparallel	Parallel	Beta-Turn	Random Coil	Total Sum
WT	16.6%	41.3%	14.5%	21.5%	44.0%	137.9%
E475A	16.6%	41.3%	14.5%	21.5%	44.0%	137.9%
H434A	16.6%	41.3%	14.5%	21.5%	44.0%	137.9%
H476A	16.6%	41.3%	14.5%	21.5%	44.0%	137.9%
R386A	16.6%	41.3%	14.5%	21.5%	44.0%	137.9%
N443A	16.6%	41.3%	14.5%	21.5%	44.0%	137.9%
H441A	16.6%	41.3%	14.5%	21.5%	44.0%	137.9%
T390A	16.6%	41.3%	14.5%	21.5%	44.0%	137.9%
K401A	16.6%	41.3%	14.5%	21.5%	44.0%	137.9%

Supplementary Fig. S12

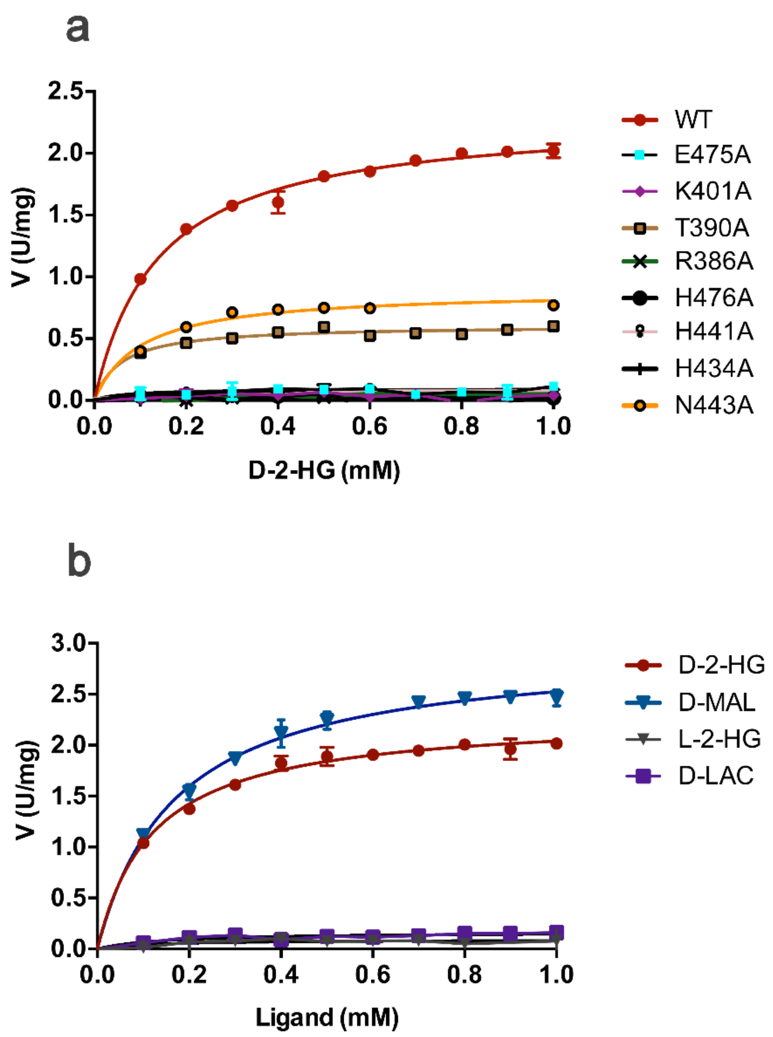

Supplementary Fig. S13

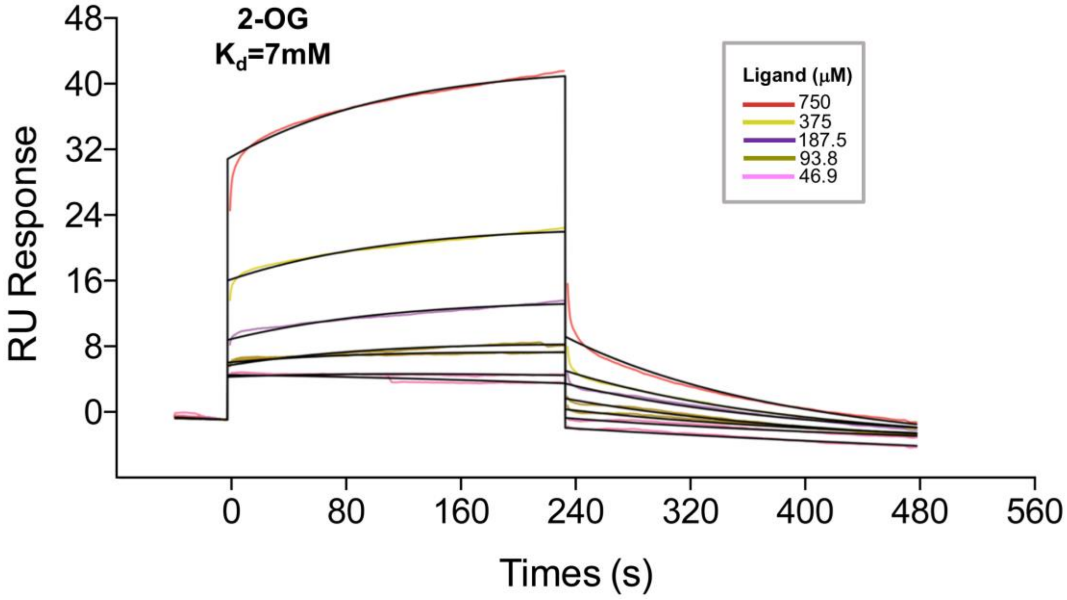

Supplementary Fig. S14

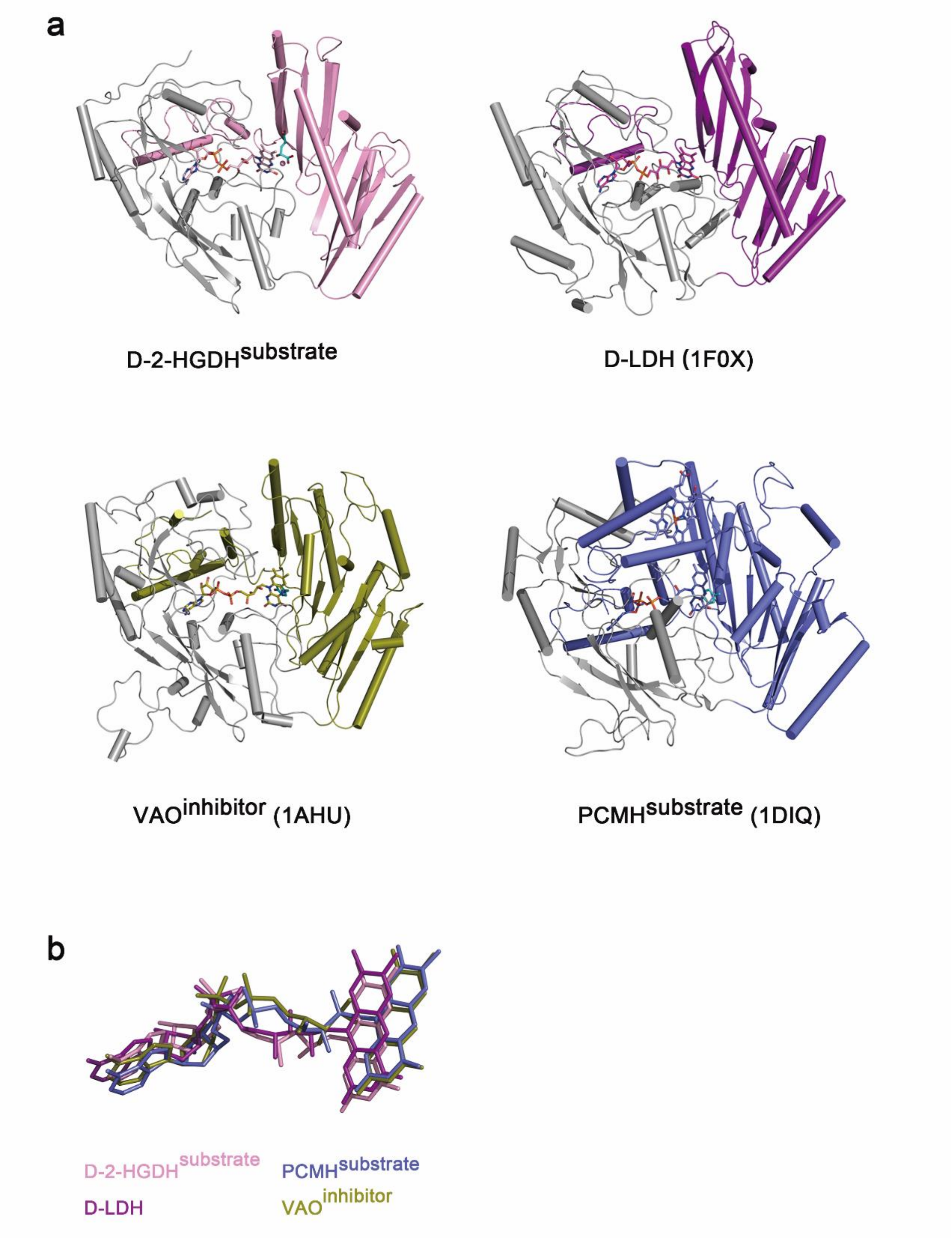

Supplementary Table S1. Kinetic parameters of wild-type and mutant D-2-HGDH towards D-2-HG and wild-type D-2-HGDH towards D-MAL

Ligand	D-2-HGDH	Specific activity ($U \cdot mg^{-1}$)	V_{max} ($U \cdot mg^{-1}$)	K_m (mM)	k_{cat} (min^{-1})	k_{cat}/K_m ($min^{-1} \cdot mM^{-1}$)
D-2-HG	WT	2.02±0.04	2.29±0.03	0.12+0.01	119±1.56	992
	R386A	0.03±0.01	ND	ND	ND	ND
	T390A	0.60±0.02	0.61±0.01	0.06±0.01	31.7±0.52	528
	K401A	0.04±0.02	ND	ND	ND	ND
	H434A	0.04±0.03	ND	ND	ND	ND
	H441A	0.08±0.01	ND	ND	ND	ND
	N443A	0.77±0.01	0.89±0.02	0.10±0.03	46.4±1.04	464
	E475A	0.08±0.01	ND	ND	ND	ND
	H476A	0.02±0.01	ND	ND	ND	ND
D-MAL	WT	2.52±0.05	2.96±0.05	0.17±0.01	154±2.60	906
D-LAC	WT	0.16±0.01	0.17±0.01	0.15±0.05	8.84±0.52	58.9
L-2-HG	WT	0.06±0.01	ND	ND	ND	ND

ND, not detected.

Supplementary Table S2. Comparison of the apo D-2-HGDH structure with the ligand-bound D-2-HGDH structures

Structure	RMSD (Å)	Aligned Cα atoms
D-2-HGDH$^{FAD+Zn+D\text{-}2\text{-}HG}$	0.45	466
D-2-HGDH$^{FAD+Zn+D\text{-}MAL}$	0.43	466
D-2-HGDH$^{FAD+Zn+D\text{-}LAC}$	0.44	466
D-2-HGDH$^{FAD+Zn+L\text{-}2\text{-}HG}$	0.47	466
D-2-HGDH$^{FAD+Zn+2\text{-}OG}$	0.44	466

Supplementary Table S3. Potential functional roles of the disease-associated mutations of D-2-HGDH

Mutation	Residual activity (WT=100%) (This work)	Residual activity (WT=100%)[3]	Structure location	Possible impact	Pathogenicity
**Type I D-2-HGA**					
**FAD-binding domain**					
S109W[3-5]	11	3	β1-α3 loop	Protein folding and stability	Pathogenic
L124P[3]	0	0	α3-β2 loop	Protein folding and stability	Pathogenic
A125T[3,4,6]	4	6	α3-β2 loop	Protein folding and stability	Pathogenic
N127K[3-5]	0	0	β2	FAD binding	Pathogenic
G131V[3-5]	0	0	β2-β3 loop	FAD binding	Pathogenic
I147S[3-5,7]	2	0	β3	FAD binding	Pathogenic
M153V[3-5]	12	2	β3-β4 loop	Protein folding and stability	Pathogenic
M153T[3-5,8]	15	0	β3-β4 loop	Protein folding and stability	Pathogenic
Q169P[3]	6	0	β5	Protein folding and stability	Pathogenic
A170E[3]	4	0	β5-α4 loop	FAD binding	Pathogenic
C172Y[3-5]	22	2	β5-α4 loop	Protein folding and stability	Pathogenic
P189L[3-5]	2	2	α4-α5 loop	Protein folding and stability	Pathogenic
I200T[3]	0	1	α5	FAD binding	Pathogenic
A205V[3-5]	2	40	α5	FAD binding	Pathogenic
A231V[3-5]	9	45	β6-β7 loop	Protein folding and stability	Pathogenic
G233S[3-5]	85	5	β7	Protein folding and stability	Non-pathogenic
C272R[3]	5	0	β8-β9 loop	Protein folding and stability	Pathogenic

**Substrate-binding domain**					
E311K[3]	1	0	β10	Substrate binding	Pathogenic
E333K[3]	73	57	α8-β11 loop	Protein folding and stability	Non-pathogenic
D375Y[3-5,9]	41	17	β12-α10 loop	Protein folding and stability	Likely pathogenic
A392G[3]	65	59	α11	Protein folding and stability	Non-pathogenic
V399M[3-5]	27	25	β13	Protein folding and stability	Pathogenic
R419H[3-5]	44	37	α12	Protein folding and stability	Likely pathogenic
A426T[3-5]	94	94	α12-β14	Protein folding and stability	Non-pathogenic
G436V[3]	69	87	β14-β15	Substrate binding	Non-pathogenic
N439D[3-5,10]	28	36	β15	Substrate binding	Pathogenic
V444A[3-5,7]	15	23	β15	Protein folding and stability	Pathogenic
A446V[3-5]	33	67	β15	Protein folding and stability	Likely pathogenic
L453F[3]	84	68	α13	Protein folding and stability	Non-pathogenic
A474V[3]	3	1	α14-η4 loop	FAD binding	Pathogenic
G477R[3-5]	3	0	α14-η4 loop	FAD binding	Pathogenic
**DLBCL**					
A208T[11]	2	not reported	α5-η1 loop	FAD binding	Pathogenic
R212W[11]	2	not reported	α5-η1 loop	Protein folding and stability	Pathogenic
R421H[11]	86	not reported	α12	Protein folding and stability	Non-pathogenic
A426T[11]	94	94	α12-β14	Protein folding and stability	Non-pathogenic

Supplementary Table S4. Structural comparison of D-2-HGDH with other representative members of the VAO/PCMH family

Structure	PDB code	RMSD (Å)	Aligned Cα atoms	Sequence identity (%)	Reference
D-LDH	1F0X	2.0	382	18.3	12
$VAO^{inhibitor}$	1AHU	2.6	388	15.2	2
$PCMH^{substrate}$	1DIQ	2.6	393	13.5	13

References

1. Gygli, G., Lucas, M.F., Guallar, V. & van Berkel, W.J.H. The ins and outs of vanillyl alcohol oxidase: Identification of ligand migration paths. *PLoS Comput. Biol.* **13**, e1005787 (2017).
2. Mattevi, A. *et al.* Crystal structures and inhibitor binding in the octameric flavoenzyme vanillyl-alcohol oxidase: the shape of the active-site cavity controls substrate specificity. *Structure* **5**, 907-20 (1997).
3. Pop, A. *et al.* D-2-hydroxyglutaric aciduria Type I: Functional analysis of D2HGDH missense variants. *Hum. Mutat.* (2019).
4. Kranendijk, M., Struys, E.A., Salomons, G.S., Van der Knaap, M.S. & Jakobs, C. Progress in understanding 2-hydroxyglutaric acidurias. *J. Inherit. Metab. Dis.* **35**, 571-87 (2012).
5. Kranendijk, M. *et al.* Evidence for genetic heterogeneity in D-2-hydroxyglutaric aciduria. *Hum. Mutat.* **31**, 279-83 (2010).
6. Ali Pervaiz, M. *et al.* Co-morbidity of Sanfilippo syndrome type C and D-2-hydroxyglutaric aciduria. *J. Neurol.* **258**, 1564-5 (2011).
7. Struys, E.A. *et al.* Mutations in the D-2-hydroxyglutarate dehydrogenase gene cause D-2-hydroxyglutaric aciduria. *Am. J. Hum. Genet.* **76**, 358-60 (2005).
8. Haliloglu, G. *et al.* Peripheral neuropathy in a patient with D-2-hydroxyglutaric aciduria. *J. Inherit. Metab. Dis.* **32 Suppl 1**, S21-5 (2009).
9. Misra, V.K. *et al.* Phenotypic heterogeneity in the presentation of D-2-hydroxyglutaric aciduria in monozygotic twins. *Mol. Genet. Metab.* **86**, 200-5 (2005).
10. Struys, E.A. *et al.* Mutations in phenotypically mild D-2-hydroxyglutaric aciduria. *Ann. Neurol.* **58**, 626-30 (2005).
11. Lin, A.P. *et al.* D2HGDH regulates alpha-ketoglutarate levels and dioxygenase function by modulating IDH2. *Nat. Commun.* **6**, 7768 (2015).
12. Dym, O., Pratt, E.A., Ho, C. & Eisenberg, D. The crystal structure of D-lactate dehydrogenase, a peripheral membrane respiratory enzyme. *Proc. Natl. Acad. Sci. USA* **97**, 9413-8 (2000).
13. Cunane, L.M. *et al.* Structures of the flavocytochrome *p*-cresol methylhydroxylase and its enzyme-substrate complex: gated substrate entry and proton relays support the proposed catalytic mechanism. *J. Mol. Biol.* **295**, 357-74 (2000).
